# Reduction of the Thermal Conductivity of Polyurethanes by Fluorination: Impact of Crystallinity, Atomic Density, and Sound Velocity

**DOI:** 10.1002/anie.202503497

**Published:** 2025-04-25

**Authors:** Jingyi Zhou, Chen Chen, Jinchi Sun, Thomas R. Fielitz, Weijun Zhou, David G. Cahill, Paul V. Braun

**Affiliations:** ^1^ Department of Materials Science and Engineering Grainger College of Engineering University of Illinois Urbana−Champaign Urbana IL 61801 USA; ^2^ Beckman Institute for Advanced Science and Technology University of Illinois Urbana−Champaign Urbana IL 61801 USA; ^3^ Department of Chemistry University of Illinois Urbana−Champaign Urbana IL 61801 USA; ^4^ Materials Research Laboratory University of Illinois Urbana−Champaign Urbana IL 61801 USA; ^5^ The Dow Chemical Company Midland MI 48667 USA; ^6^ The Dow Chemical Company Lake Jackson TX 77566 USA

**Keywords:** Crystallinity, Fluorinated polymers, Microstructure, Sound velocity, Thermal conductivity

## Abstract

The intrinsic thermal conductivity (Λ) of polymers ranges between 0.13 W m^−1^K^−1^ in amorphous polyvinyl chloride to 60 W m^−1^K^−1^ in ultrahigh molecular weight polyethylene. Increasing the amorphous content of polymers to further lower Λ is insufficient as this approach reaches a practical limit at approximately 0.15 W m^−1^K^−1^. Inspired by the low Λ and low speed of sound of fluorinated liquids, we explored whether this behavior in liquids can be extended to polymers. We synthesized seven partially fluorinated (9%–17% atomic fraction F) and ten conventional polyurethanes. Fluorinated polyurethanes exhibit a reduction in Λ up to 50% compared to their nonfluorinated counterparts. Microstructural analysis revealed that the fluorinated polyurethanes exhibited reduced crystallinity and increased molecular spacing. Furthermore, we observed a decreased speed of sound in fluorinated polymers by forced Brillouin scattering via a new analysis method that captures weak signals from highly scattering semicrystalline polymers. The lowest thermal conductivity, 0.13 W m^−1^K^−1^ at room temperature, was observed in polyurethane synthesized from 2,2,3,3,4,4,5,5‐octafluoro‐1,6‐hexanediol (16F) and isophorone diisocyanate (IPDI). Our study provides deeper insights into the relationship between Λ, microstructure, and chemical structure, paving the way to rational design of polymers with thermal conductivity below the lowest limit of conventional amorphous polymers.

## Graphical abstract

This study provides a rich database of structure–property relationships that will help guide future work in designing polymers with low intrinsic thermal conductivity. Fluorinated polymers are widely utilized in industry for their outstanding hydrophobicity, chemical resistance, and heat resistance. Our study provides insights that fluorinated polymers have generally lower thermal conductivity, offering benefits for industry where thermal insulation is desired. This study provides an important step in the validation of computational studies and advances the development of machine learning models for the thermal conductivity of polymers.

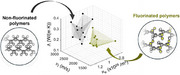

## Introduction

The low thermal conductivity (Λ) of polymers plays a crucial role in thermally insulating materials used for temperature regulation and energy efficiency with the thermal conductivity of most polymers falling in the range of 0.15 to 0.30 W m^−1^K^−1^.^[^ ^1^, ^2^, ^3^ ^]^ Engineering the intrinsic Λ of polymers has become a prominent topic, where intrinsic Λ is defined as the inherent ability of a material to conduct heat without, for example, resorting to the formation of composites or foams. Most research on engineering Λ of polymers has focused on enhancing Λ through, for example, chain alignment.^[^ ^4^ ^]^ By improving chain alignment, ultrahigh molecular weight polyethylene (UHMPE) was reported to have high thermal conductivity, exceeding 60 W m^−1^K^−1^.^[^ ^5^ ^]^ In contrast, attempts to reduce polymer Λ encounter an empirical lower limit of approximately 0.15 W m^−1^K^−1^ by simply reducing the crystallinity and the strength of interchain interactions.^[^ ^6^, ^7^, ^8^ ^]^

To date, the lowest intrinsic Λ observed in solid materials is 0.05 W m^−1^K^−1^ in the functionalized fullerene, [6,6]‐phenyl‐C61‐butyric acid *n*‐butyl ester.^[^ ^9^ ^]^ However, fullerene derivatives are challenging to functionalize and expensive for large‐scale applications.^[^ ^10^ ^]^ The most common method to form thermally insulating polymers involves filling polymers with gas to create foams, which can reduce Λ to approximately 0.03 W m^−1^K^−1^, but at the cost of mechanical stiffness and strength.^[^ ^11^ ^]^ There is limited research on developing a general strategy to reduce the intrinsic Λ of polymers, a crucial factor in creating better thermal insulators. A reduction in intrinsic Λ not only aids in engineering bulk polymers with low Λ and high mechanical strength but would also enable polymer foams (where the solid thermal conductivity comprises 15%–30% of the overall Λ) and polymer aerogels (solid is >30% of the overall Λ, due to Knudsen effect) with further enhanced thermal insulation properties.^[^ ^12^, ^13^, ^14^ ^]^

Recently, we observed that several fluorinated liquids exhibit extremely low Λ in comparison to chemically similar nonfluorinated liquids at the same molar density due to lower speed of sound (Figure S1).^[^ ^15^, ^16^, ^17^ ^]^ According to the Bridgman formula, the coefficient (γ) of Λ for liquids ($\Lambda =k_{B}\;\gamma$) can be predicted via $\gamma =\frac{2c_{s}}{\Delta^{2}}$, where *c*_s_ is the liquid sound velocity, and Δ is related to the molar molecular density (*n*) via Δ  = (*N*_A_ *n*)^−1/3^ , *N*_A_ is the Avogadro's number.^[^ ^18^ ^]^ 2‐(Trifluoromethyl)‐3‐ethoxydodecafluoro‐hexane (commercially available from 3 M as Novec HFE 7500), a fluorinated liquid, has a molecular density of 4 mol L^−1^ and Λ of 0.07 W m^−1^K^−1^ at 300 K, whereas Λ = 0.14 W m^−1^K^−1^ for dodecane liquid. As the molecular densities of these two liquids is similar, the factor of two reduction in thermal conductivity can be attributed to the lower sound velocity in fluorinated liquids. Specifically, the sound velocity in *n*‐dodecane is 1260 m s^−1^ and in HFE 7500 is 670 m s^−1^.^[^ ^19^, ^20^ ^]^ Although the Bridgman equation applies accurately only to liquid molecules with small and rigid molecular structure, experiment results show that compared to the nonfluorinated liquid counterparts, a lower speed of sound and a larger intermolecular distance were observed in fluorinated liquids.^[^ ^18^, ^21^ ^]^

Inspired by the behavior of fluorinated liquids, we introduced fluorine into precursor materials for a series of polyurethane (PU) formulations. By comparing various chemical structures, we investigated the effects of fluorination in polymer crystallization, molecular packing, sound velocity, and coefficient of thermal expansion. We found that polymers containing fluorine exhibit lower crystallinity, reduced atomic density (*n*_a_), and a decreased speed of sound (*v*_l_). These factors, arising from the large atomic size and high electronegativity of fluorine, collectively contribute to reduced Λ. Furthermore, based on variations in chemical structures, we found that fluorine added onto aliphatic chains is more effective in reducing *Ʌ* compared to fluorine added to aromatic rings. The lowest Λ observed in this study is 0.13 W m^−1^K^−1^ in a polyurethane synthesized from 2,2,3,3,4,4,5,5‐octafluoro‐1,6‐hexanediol (16F) and isophorone diisocyanate (IPDI). Thus, by incorporating fluorine into polyurethanes, the 0.15 W m^−1^K^−1^ limit for conventional amorphous polymers was surpassed. To further reduce the thermal conductivity of fluorinated polyurethanes, structures with higher fluorine content are promising candidates.^[^ ^22^ ^]^ Introducing chlorine also has the potential to reduce polymer thermal conductivity. However, chlorinated diols are not widely available and chlorinated polymers are generally less stable at elevated temperature. Polyvinyl chloride degrades at 250 °C, whereas polyvinyl fluoride degrades at 350 °C in air.^[^ ^23^, ^24^ ^]^ Therefore, we focus on fluorinated polyurethanes in this study.

This study provides an initial database that documents crystallinity, entropy change, thermal conductivity, density, and sound velocity of low thermal conductivity polymers. We anticipate that our results will be a valuable resource for theory, experiment, and machine learning aimed at designing new polymers with low thermal conductivity.

## Results and Discussion

### Polyurethane Structure Database

**Figure 1:**
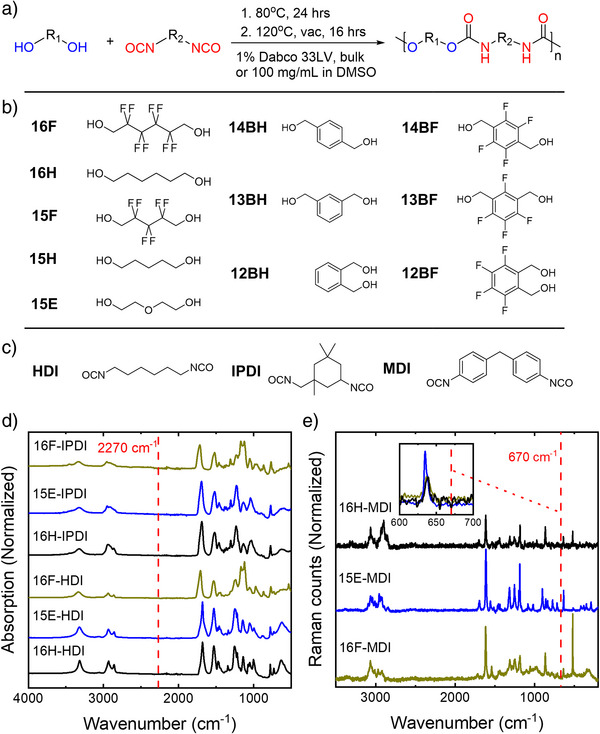
PU synthesis and structural database. a) PU synthesis conditions employed in this study. b) Diols and c) isocyanates structures involved in this study. d) Attenuated total reflectance (ATR) spectrum of representative PUs synthesized from bulk condensation. The red line indicates the absorbance of the isocyanate group. e) Representative FT‐Raman spectrum of PU synthesized from solution, excited at 1064 nm with a laser intensity of 0.2 W. The PU powder was placed on a silicon substrate, and the background was extracted via polynomial fitting. The red line indicates the most intense peak position of DMSO at 670 cm^−1^. ATR and FT‐Raman of other synthetic PUs were provided in Figures S7 and S8.

Details for the instruments, materials, and protocols are provided in Supporting Information. The product PU is labeled using a combined abbreviation of the diols and isocyanates. For example, the PU produced from 16F and HDI, as shown in Figure 1b,c, is designated as 16F‐HDI. The produced PUs were analyzed using attenuated total reflectance (ATR) infrared spectroscopy to confirm the complete consumption of isocyanate, which has a peak at approximate 2270 cm^−1^, indicated by the red line in Figure 1d.^[^ ^25^ ^]^

FT‐Raman was further applied to PUs synthesized from solution, to confirm the complete removal of DMSO, which exhibits a peak at 670 cm^−1^, shown as the red line in Figure 1e.^[^ ^26^ ^]^ Representative TGA spectra are provided in Figure S5.

The thermal conductivity of amorphous polymers is found to be independent of chain length for molecular weight larger than 10^3^ g mol^−1^.^[^ ^6^ ^]^ To evaluate if the molecular weights of our synthesized polyurethanes exceed this threshold, the molecular weight of the three polyurethanes synthesized from the least reactive isocyanate, IPDI, was measured by GPC. We assume that the chain lengths of polyurethanes synthesized from IPDI will be a lower bound. The GPC data and analysis are included in the Supporting Information as Figure S6 and Table S1. All three PUs synthesized from IPDI have chain lengths exceeding 10^3^ g mol^−1^. Although this study did not explore highly drawn polymers, it is worth noting that in semicrystalline polymers with high draw ratios, thermal conductivity increases with increasing molecular weight.^[^ ^27^, ^28^ ^]^

### Fluorinated Polymers Have Reduced Crystallinity

The morphologies of representative PUs were characterized using three distinct methods tailored to a wide range of microstructural length‐scales. Crystal interchain distances was analyzed through wide angle X‐ray scattering (WAXS), probing lengths ranging from 0.3 to 3 nm. Microphase separation was characterized by small angle X‐ray scattering (SAXS), probing lengths ranging from 3 to 30 nm. Larger‐scale crystalline structures were examined using polarized optical microscopy (POM), capable of resolving features from a few micrometers up to several millimeters.^[^ ^29^ ^]^

**Figure 2:**
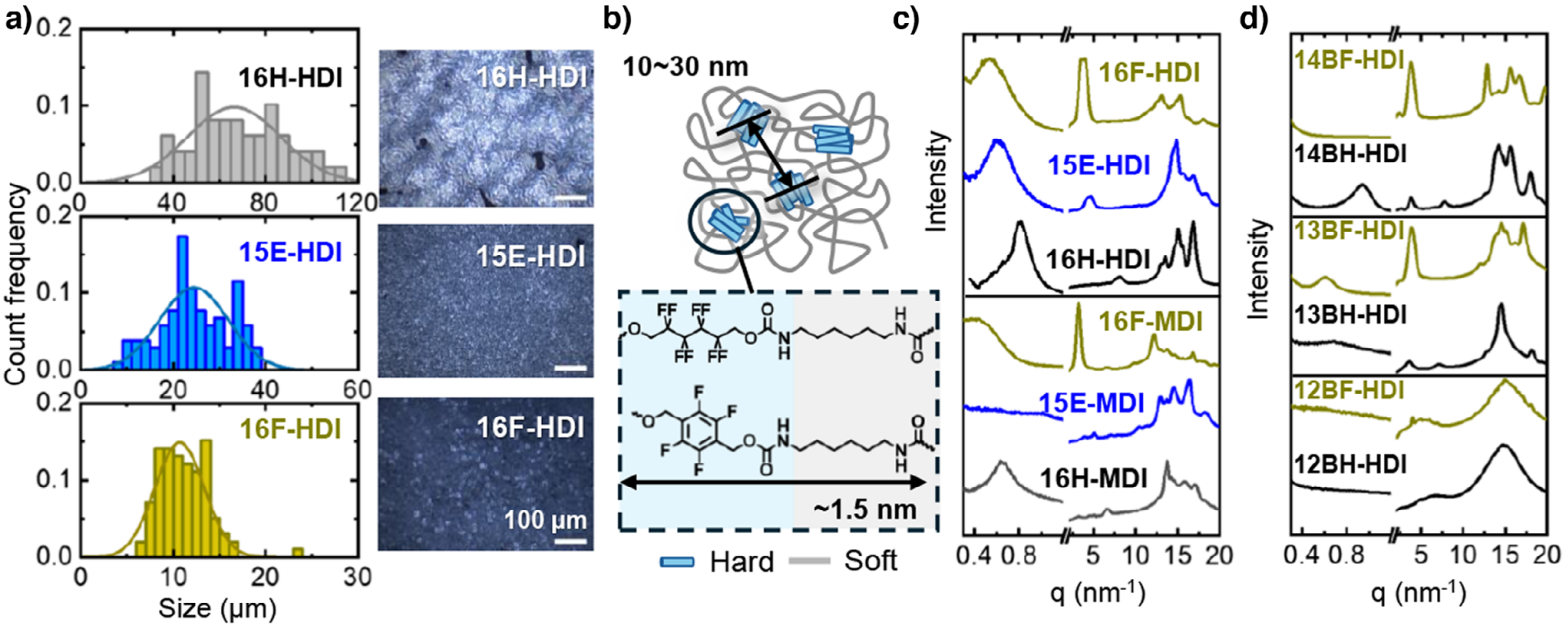
Crystallinity in synthesized PUs. a) Crystallite size distribution in PUs and representative polarized microscope images (POM) obtained using a 10× objective lens at 90° crossed polarizers. Scale bar: 100 µm. The light intensity was maintained the same and data collected using reflection light. The bin size of 16H‐HDI is 5 µm, of 15E‐HDI is 2 µm, and of 16F‐HDI is 1 µm. b) Illustration of microphase separation and representative repeat unit length in PUs. Wide angle X‐ray scattering (WAXS) combined with small angle X‐ray scattering (SAXS) for c) PUs synthesized from diols 16F, 15E, and 16H with isocyanates HDI or MDI. The intensity was normalized; d) PUs synthesized from aromatic diols with isocyanate HDI.

We first compared three PUs synthesized from linear diols and linear isocyanate HDI, named 16F‐HDI, 15E‐HDI, and 16H‐HDI. The macroscale crystal size was measured using POM during crystallization. The statistically averaged spherulite diameter was 70 µm for 16H‐HDI, 20 µm for 15E‐HDI, and 10 µm for 16F‐HDI (Figure 2a). The introduction of fluorine atoms reduced both the spherulite diameter observed under POM and the optical contrast between the background and the crystal domains. At the smaller‐scale characterized by SAXS, as shown in Figure 2c, we observed a peak (∼0.4–0.8 nm^−1^) indicating microphase separation between hard and soft segments. The position of this peak is comparable to the range of peak positions, 0.4–1.0 nm^−1^, typically reported for conventional PUs.^[^ ^30^, ^31^ ^]^ For fluorinated 16F‐HDI, the peak is shifted to lower scattering vector, 0.5 nm^−1^, compared to other PUs, reflecting the larger distance between hard segment aggregations (Figure 2b).

In WAXS data, we observed a strong peak in fluorinated 16F‐HDI at approximately 4 nm^−1^ (Figure 2c). We propose that this peak arises from density fluctuations caused by fluorine atoms. We estimated the length of PUs repeat units using the PubChem database and Pymol software by measuring and summing the averaged distance of molecular chain ends of energy favored configuration of the monomers (Figure S9). The length of the PU repeat unit calculated in this way is 1.5 nm. This value is the same as the length‐scale of 1.5 nm given by the center position of the WAXS peak through $d=\frac{2\pi }{q}$ (Figure 2b) to within the experimental uncertainties.^[^ ^32^ ^]^

The crystallinity of PUs was calculated by integrating the baseline corrected WAXS peaks from 10 to 20 nm^−1^, according to the equation *X*_c,WAXS_ = *I*_c_ /(*I*_c_ + *I*_a_), where *I*_c_ is the integral of the intensity of the crystalline peaks, and *I*_a_ the integral of the intensity of the amorphous background. The method employed to measure the crystal fraction was validated by comparing the melting enthalpy from differential scanning calorimetry (DSC) with standard polymers, as shown in Figures S10 and S11.^[^ ^33^ ^]^ The crystallinity of 16F‐HDI was reduced to 31%, compared to 16H‐HDI, which has a higher crystallinity of 68% (Figure S12). This indicates that the introduction of fluorine can decrease the crystallinity of polymers in chemically similar structures (Figures S11 and S12). The crystallinity was also characterized in commercially available polymers, where crystallinity is HDPE (61%), PTFE (54%), and PVDF (54%). However, the difference is not as pronounced as that observed in fluorinated PUs. The coefficient of thermal expansion however does not exhibit a clear trend, where 16H‐HDI is 120 x 10^−6^ per K, 15E‐HDI is 70 x 10^−6^ per K, and 16F‐HDI is 90 x 10^−6^ per K, as summarized in Table S2.

In addition to the aliphatic polymers discussed above, we introduced aromatic structures to further investigate the influence of chemical composition on microstructure. Solution polymerization was employed to synthesize aromatic PUs using the same aliphatic diols with an aromatic isocyanate MDI to reduce the reaction rate and enable the formation of homogenous PUs, designated as 16H‐MDI, 15E‐MDI, and 16F‐MDI. The feature of larger microphase separation length scale in SAXS was consistently observed in fluorinated 16F‐MDI, whereas no microphase separation was detected in this range for 15E‐MDI (Figure 2c). However, when MDI is used as the isocyanate, the crystallinity of these three PUs is nearly identical: 16H‐MDI (39%), 15E‐MDI (35%), and 16F‐MDI (38%).

We considered the question if the position of the fluorine atoms in the aromatic ring influences the microstructure by studying fluorinated aromatic diols. As shown in Figure 2d, 12BF and 12BH formed nearly fully amorphous PUs with no clear evidence of ordered microphase separation observed, which could be partially attributed to the sterically constrained position of urethanes that makes it difficult to form interchain hydrogen bond (Figure S13). It has been reported that hydrogen bonding influences the carbonyl stretching frequency, causing the FT‐IR absorption of the carbonyl group shift to lower frequencies by approximately 20–30 cm^−1^.^[^ ^34^ ^]^ This effect was also observed in ATR spectra, as shown in Figure S13, though the shift is smaller, ranging from 4 to 7 cm^−1^. Additionally, in SAXS measured phase separation, fluorinated PUs generally have an increased distance between hard segment aggregates in their chemical counterparts (Figure 2d). Notably, 14BF‐HDI exhibits no discernible peak at low scattering vector, 0.2–1 nm^−1^, but it generated higher intensity counts near the beam center in SAXS. In other words, we are unable to observe a microphase separation peak in 14BF‐HDI due to the lower limit to the scattering vector that can be probed in our SAXS instrument. The crystallinity was less affected by the introduction of fluorine, which we measured as 13BH‐HDI (31%) and 13BF‐HDI (29%) as well as14BH‐HDI (48%) and 14BF‐HDI (40%).

Another effect related to chemical structure is the odd–even effect. PUs synthesized from diols with an odd number of carbons were named as 15F‐HDI and 15H‐HDI. We observe that only 15H‐HDI shows crystallization in polarized optical microscopy, whereas 15F‐HDI has no apparent crystallization (Figure S14). However, for even carbon numbers both fluorinated and nonfluorinated PUs show crystalline regions. This behavior is consistent with WAXS and SAXS analysis, where only amorphous peaks or peaks due to fluorine density fluctuations were observed (Figure S14). This indicates that in this type of structure, fluorinated diols with an odd number of carbon atoms will mostly form amorphous polymers. In contrast, without fluorine, crystallinity is not significantly affected and remains at approximately 50% for both odd and even nonfluorinated PUs.

In this study, we considered fluorinated polymers where the fluorination is either complete or the arrangements of fluorine and hydrogen atoms are well‐ordered. PTFE and PVDF provide a point of comparison. The crystallinity of PTFE and PVDF are both approximately 50%. A disordered arrangement of fluorine substitution will tend to reduce crystallinity. For example, atactic polyvinyl fluoride (PVF) exhibits a minimum crystallinity close to 20% when polymerized at 100 °C.^[^ ^35^ ^]^ We have not explored the effects of disordered arrangements of fluorine substitutions in this study although reduced crystallinity tends to reduce thermal conductivity.

### Fluorinated Polymers Have Lower Atomic Density

**Figure 3:**
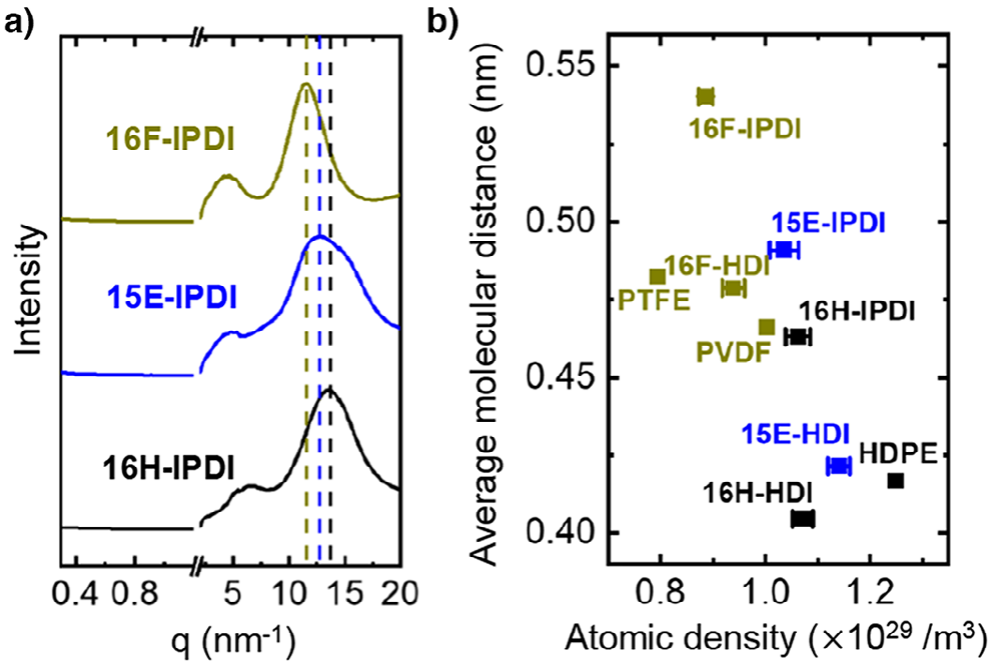
Molecular distance characterization of PUs. a) Combined WAXS and SAXS for complete amorphous PUs. b) Average molecular distance calculated from Gaussian fitted amorphous peak center for representative PUs and standard polymers.

In addition to the trends observed in crystallinity, we also noted a shift in the amorphous peaks of fluorinated polymers toward larger distances. When using IPDI as the isocyanate to form 16F‐IPDI, 15E‐IPDI, and 16H‐IPDI, all resulting PUs are amorphous due to the steric structure of IPDI (Figures 3a and S15). We observed that the center of the peak that is characteristic of amorphous state packing (∼12 nm^−1^) shifted due to the introduction of fluorine. Specifically, 16F‐IPDI exhibited the dominant amorphous state spacing peaks at 12 nm^−1^, shifted to lower scattering vector by 15% compared to 16H‐IPDI at 14 nm^−1^. This feature was consistent with what we observed for the center of the amorphous peaks in 16H‐HDI, 15E‐HDI, and 16F‐HDI.

We used a Gaussian function to fit the center position of the amorphous peak *q*_a_ in PUs and define this distance as average molecular distance by $=\frac{2\pi }{q_{a}}$. This distance includes both intermolecular and intramolecular short range order.^[^ ^36^ ^]^ The atomic density was calculated according to the equation $n_{a}=\frac{\rho N_{A}}{\mathrm{MN}}$, where *ρ* is the density measured by pycnometer, *M* is the molecular weight of the repeat unit, and *N* represents the number of atoms in a single repeat unit. We plot the average molecular distance as a function of atomic density in Figure 3b. When comparing polymers with chemically similar structures, fluorinated polymers typically exhibit larger average molecular distances in their amorphous state. We attribute this effect to the steric effect of fluorine atoms and flexibility of chains. However, when fluorine is added to an aromatic ring, we do not observe larger average molecular distance (Figure 2d).

### Reduced Thermal Conductivity in Fluorinated Polymers

The thermal conductivity (Λ) of the PUs described above, along with representative conventional polymers, was measured by time‐domain thermoreflectance (TDTR) and displacement‐thermo‐optic phase spectroscopy (D‐TOPS). TDTR is mostly sensitive to thermal effusivity ($\sqrt{\Lambda C}$) and through‐plane Λ, whereas D‐TOPS is mostly sensitive to thermal diffusivity ($\frac{\Lambda }{C}$) in the in‐plane direction (*C* is the volumetric heat capacity). Therefore, if the polymer does not exhibit anisotropy, analyzing the TDTR and D‐TOPS measurements using the volumetric heat capacity *C* measured by DSC should produce consistent values for Λ. By applying the heat capacity measured from DSC, we observed that TDTR and D‐TOPS measurements of Λ agreed within approximately 10%, i.e., Λ measured by both methods lies on the equivalent line, which is consistent with our observation of isotropic X‐ray scattering signal (Figures S16 and S17).

In D‐TOPS, the pump laser is frequency‐modulated and heats the transducer that is deposited on the frontside of the sample. (We previously referred to this measurement as “front‐side frequency‐domain probe beam deflection.”^[^ ^37^, ^38^, ^39^ ^]^) The measurement geometry of D‐TOPS is, therefore, identical to that of TDTR. The optical layout of our instruments is illustrated in Figures S2 and S3. The sensitivity of each method was calculated based on sample geometry (Figure S18). In what follows, we calculated the average thermal conductivity as Λ = $\frac{1}{3}\Lambda_{z}+\frac{2}{3}\Lambda_{r}$, accounting for the two in plane directions (*x,y*) measured by D‐TOPS and one through‐plane (*z*) direction measured by TDTR. In the limit of small anisotropy, the mean of the components of the thermal conductivity tensor is a good approximation to the orientationally‐averaged thermal conductivity.^[^ ^40^ ^]^ The systematic error that propagates from uncertainty in the volumetric heat capacity is also reduced by taking this average.

**Figure 4:**
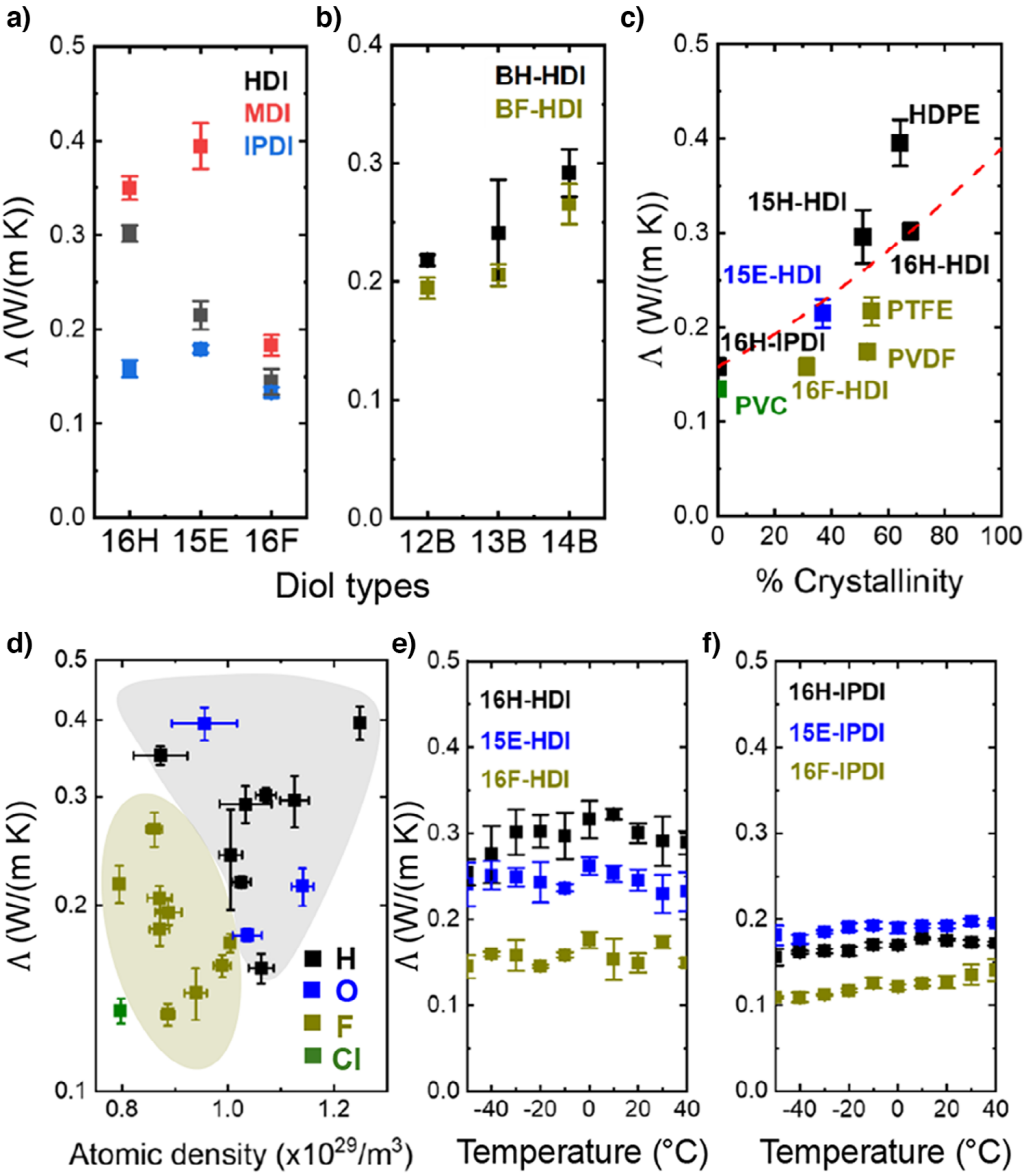
Thermal conductivity (Λ) of synthetic PUs and standard polymers. In the following figures, Λ represents the averaged thermal conductivity, calculated as $\frac{1}{3}\Lambda_{z}+\frac{2}{3}\Lambda_{r}$. $\Lambda_{r}$, in‐plane thermal conductivity measured by D‐TOPS. $\Lambda_{z}$, through‐plane thermal conductivity measured by TDTR. The errors were propagated from the uncertainties of the two methods. A summary of Λ values is provided in Table S2. a) and b) Thermal conductivity of synthetic PUs. c) Relationship between crystallinity and thermal conductivity; crystallinity was calculated from fitting WAXS data. The red line represents the predicted thermal conductivity by effective medium theory. d) Thermal conductivity versus atomic density, where the dark yellow range presents the fluorinated polymers. H, O, F, and Cl represent polymer that has specific atoms on their backbone. Temperature‐dependent thermal conductivity of e) linear crystallized PUs and f) linear amorphous PUs. The error bar represents standard deviation.

We first compared the Λ of nine PUs synthesized using diols 16H, 15E, and 16F with isocyanates HDI, MDI, and IPDI (Figure 4a). PUs synthesized with 16F consistently exhibited lower Λ compared to other PUs with the same type of isocyanate. Compared with 16H, the 16F PUs with MDI and HDI show differences in Λ of approximately 50%; 16F‐IPDI exhibit approximately 20% lower Λ compared to 16H‐IPDI and 15E‐IPDI. Although the aromatic MDI isocyanate enhanced the Λ of PUs, the aliphatic IPDI isocyanate reduced the Λ due to its steric structure, bringing Λ of these materials closer to the empirical Λ limit of amorphous polymers.

The differences in thermal conductivity Λ between PUs synthesized from fluorinated aromatic diols (BF) and conventional aromatic diols (BH) and isocyanate HDI were small (Figure 4b). The structures of BF and BH diols are illustrated in Figure 1b. Although BF series PUs show approximately 10% lower Λ compared to the BH series, the Λ difference coming from fluorine atoms is not as significant as the difference caused by the substitution position on the aromatic ring (up to 50%). This decrease in Λ is consistent with the reduction of crystallinity from 14B to 12B.

The relatively large uncertainty in Λ for the 13BH‐HDI sample is due to a larger variability of the measurement by D‐TOPS. We attribute this problem to the fact that the glass transition of this sample is close to room temperature (Figure S19). In the analysis of the D‐TOPS data, we assume that the thermal expansion does not depend on frequency and this assumption is not always valid at temperatures where the kinetics of the *α* relaxation overlaps with the kHz modulation frequencies used in the D‐TOPS measurement. However, the Λ values were reproducible in TDTR, where segmental (*α*) relaxation is not active at the high frequency of 9.3 MHz used in the TDTR measurement.

Fluorine in the polyurethane chain can form hydrogen bonds with urethane linkages, potentially resulting in network topologies. Hydrogen bonding enhances thermal conductivity by increasing the strength of interchain interactions.^[^ ^41^, ^42^ ^]^ However, hydrogen bonding with organic fluorine is typically weak^[^ ^43^ ^]^ and the density of the urethane linkages that can serve as a hydrogen donor is small, approximately 4% of PU atomic density. Previous observations also suggested that, if hydrogen bond is treated as a cross‐linker, the cross‐link density slightly affects the thermal conductivity.^[^ ^44^ ^]^ Consequently, the influence of H⋯F interactions on intrinsic Λ of PU systems is minimal; this conclusion is supported by the lack of an obvious increase in elastic modulus of fluorinated PUs (Figure S28).

To understand the role of crystallinity in Λ, we examined the behavior of a simple effective medium composite of amorphous and crystalline regions, following our previous study and Choy's study.^[^ ^45^, ^46^, ^47^, ^48^ ^]^ We modeled the polymers as a composite of two components, an amorphous component and a crystalline component. The effective thermal conductivity Λ_eff_ was calculated using the symmetric Landauer–Bruggeman effective medium approximation:

(1) $$\begin{array}{ccc} 4\Lambda_{\mathrm{eff}} & = & \left(3f_{c}-1\right)\Lambda_{c}+\left(3f_{a}-1\right)\Lambda_{a}\\ & & +\;[\left(\left(3f_{c}-1\right)\Lambda_{c}+\;)2\left(3f_{a}-1\right)\Lambda_{a})2+8\Lambda_{a}\Lambda_{c}\right]\frac{1}{2} \end{array}$$

In this equation, *f*_c_ and *f*_a_ are the volume fractions of the crystalline and amorphous domains, respectively, and $\Lambda_{c}$ and $\Lambda_{a}$ are their thermal conductivities. We used amorphous 16H‐IPDI in this study as the empirical low Λ limit, i.e., we set $\Lambda_{a}$ to the thermal conductivity of 16H‐IPDI (0.16 W m^−1^K^−1^). We adjusted $\Lambda_{c}$ to match the model to data for 16H‐HDI. In this aliphatic polyurethane system, the effective medium prediction captures the trends in the experimental Λ except for 16F‐HDI (Figure 4c). The symmetric Landauer–Bruggeman effective medium theory adapted in this study assumes that interface resistances between the phases are negligible and that the thermal conductivities of the two phases are constant. As noted previously, these assumptions may not be well‐satisfied in semicrystalline polymers and we do not expect effective medium model to precisely predict variations in thermal conductivity with crystallinity.^[^ ^46^, ^49^ ^]^ Other models such as Maxwell model, are essentially equivalent to the Landauer–Bruggeman model when the contrast in the properties between the two phases is small.^[^ ^48^ ^]^

As Λ is often correlated with density, we investigated the relationship between atomic density and Λ as shown in Figure 4d. Fluorinated polymers have atomic densities that are approximately 20% lower than nonfluorinated polymers. We attribute this result to the larger size of fluorine relative to hydrogen. Notably, PVC has the lowest atomic density due to the presence of the even larger chlorine atoms.

To examine the trend in Λ over a broader temperature range, a temperature‐dependent study was conducted by D‐TOPS. The Λ of six linear PUs was measured over a range from −50 to 40 °C, excluding the melting temperature (Figure 4e,f). All PUs maintained a similar morphology in this range of temperatures. The sample was mounted onto a temperature‐controlled stage and placed inside a vacuum chamber with a pressure of approximately 10^−3^ Pa to thermally insulate it from the ambient. We plotted the average of three measurements on each sample in Figure 4e,f. The trend of thermal conductivity is consistent across the three measurements. Within this temperature range, the fluorinated PUs generally exhibited lower Λ compared to other PUs. Aside from the effects of lower crystallinity, the amorphous 16F‐IPDI also showed lower Λ, which indicates that low Λ of fluorinated PUs is not solely dependent on reduced crystallinity. It can be attributed to the larger molecular distance between polymer chains, whereas the 16H‐IPDI and 15E‐IPDI have reached the Λ limit of amorphous polymers, approximately 0.15 W m^−1^K^−1^.

### Slow Crystallization Dynamics and Low *Ʌ* of Fluorinated Polymer at the Same Entropy

**Figure 5:**
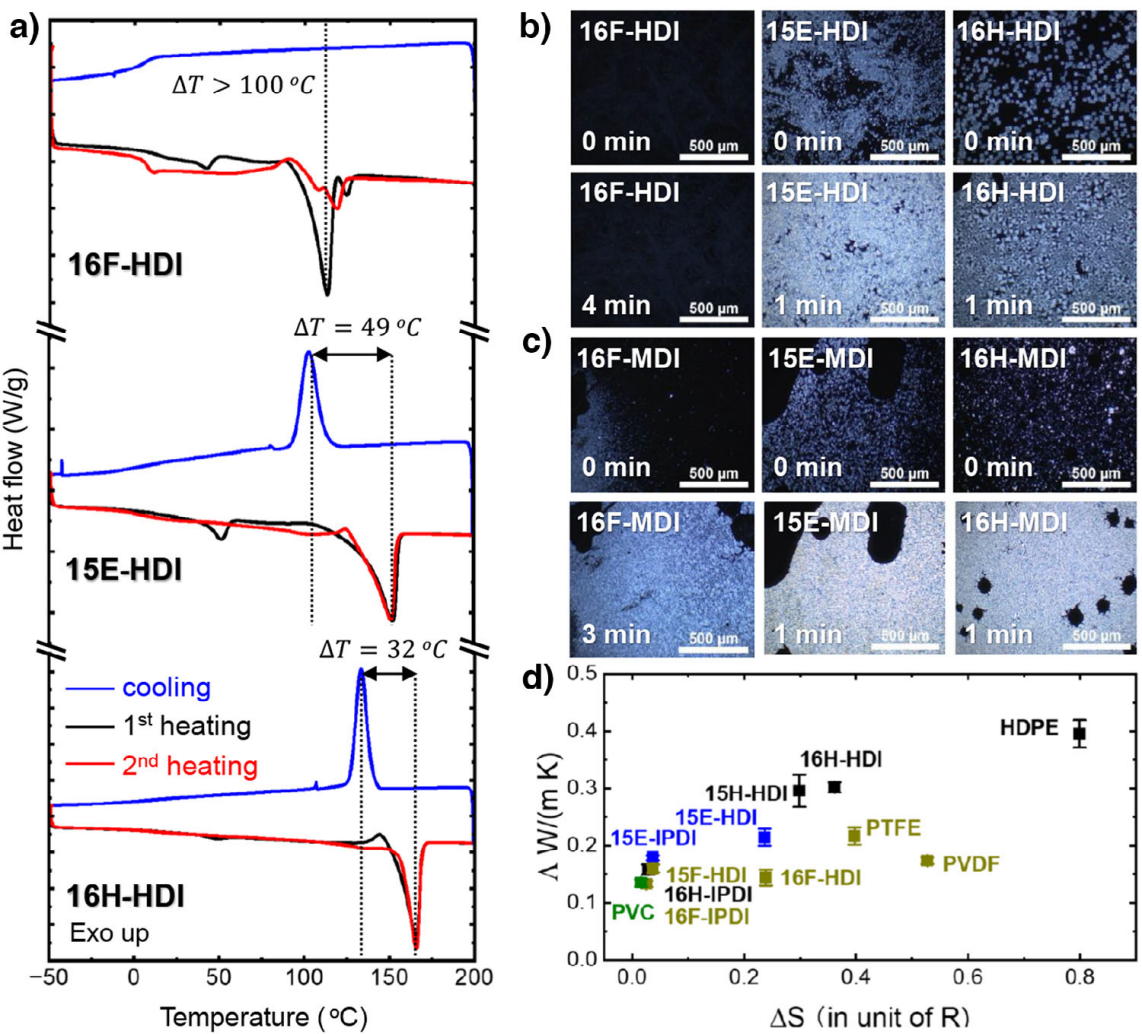
Slow crystallization rate and low thermal conductivity (Λ) at same entropy of fluorinated polymers. a) Differential scanning calorimetry (DSC) thermogram of 16F‐HDI, 15E‐HDI, and 16H‐HDI. PUs samples were sealed in TA Instruments Tzero pans without slow‐cooling and analyzed at a scanning rate of 5 °C min^−1^. b) Dynamic study using polarized optical microscope (POM) of 16F‐HDI, 15E‐HDI, and 16H‐HDI. Images were captured using a 10× objective lens with 90° polarization, at a consistent input light intensity. c) POM images of 16F‐MDI, 15E‐MDI, and 16H‐MDI with the same capturing parameters to those in (b). d) Correlation between entropy (in unit of *R*) and thermal conductivity. The *x* axis represents the change in entropy in units of *R*, where *R* is the gas constant (*R* = 8.31 J mol^−1^K^−1^).

To elucidate the causes for low Λ in fluorinated polymers, we investigated the crystallization dynamics to reveal how fluorination influences the reduction of crystallinity. We observed that in DSC data, the peak positions of melting and crystallization at same ramp rate are related to the molecular structures. In Figure S20, the crystallization peak of HDPE was suppressed by 15 °C compared to the melting peak, whereas PVDF exhibited a suppression of 30 °C and PTFE showed a suppression of 15 °C. Hence, we further studied the crystallization dynamics of synthetic PUs using DSC, as illustrated in Figure 5a. Our studies of three linear PUs with similar chemical structures indicate that the crystallization peak of 16H‐HDI was delayed by 32 °C and that of 15E‐HDI by 49 °C. In the case of 16F‐HDI, no crystallization peak was observed during cooling. We observed that 16F‐HDI underwent a glass transition at 6 °C, exhibited cold crystallization at 56 °C, and melted at 90 °C. The crystallinity increased at room temperature, as validated by a sample left overnight, which showed an increased crystal fraction (Figure S21). We also applied different cooling rates and observed that a slower cooling rate resulted in more cold crystallization (Figure S22).^[^ ^50^ ^]^

In subcooled systems, the driving force for forming nucleation can be considered as the free energy of fusion, which can be approximated by assuming heat of fusion is independent of temperature:^[^ ^51^ ^]^

(2) $$\Delta f=\Delta H_{f}-T\Delta S_{f}=\Delta H_{f}-\frac{T(\Delta H_{f})}{\left(T_{m}^{0}\right)}=\frac{\left(\Delta H_{f}\left(\Delta T\right)\right)}{\left(T_{m}^{0}\right)}$$

where Δ*H*_f_ is the heat of fusion per unit volume of polymer crystal, $T_{m}^{0}$ is equilibrium melting point of a polymer, and Δ*T* is the undercooling temperature.^[^ ^52^ ^]^ We measured $T_{m}^{0}$ using the Hoffman–Weeks method and calculated 100% crystallinity enthalpy by correlation between DSC enthalpy and WAXS crystallinity (Figures S23–S25).^[^ ^53^ ^]^ The $\Delta H_{f}/T_{m}^{0}$ factor calculated for two nonfluorinated PUs are: 16H‐HDI (0.30 J cm^−3^K^−1^) and 15E‐HDI (0.28 J cm^−3^K^−1^). At Δ*T* shown for each sample in Figure 5a, Δ*f*(16H‐HDI) ≈ 9.6 J cm^−1^ and Δ*f*(15E‐HDI) ≈ 13.7 J cm^−3^.

16F‐HDI exhibits two distinct crystalline phases, which are designated as crystal phase A and B (Figure S25c). As the crystallinity of 16F‐HDI maintained at room temperature overnight is dominated by phase A, we take the fraction of enthalpy integration as a normalization factor and calculate the driving force separately (Figure S26). Therefore, the $\Delta H_{f}/T_{m}^{0}$ for 16F‐HDI‐A is 0.31 J cm^−3^K^−1^ and for 16F‐HDI‐B is 0.31 J cm^−3^K^−1^, which is the same as A phase. As the crystallization peak of 16F‐HDI did not appear in the DSC thermogram, Δ*f*(16F‐HDI‐A) > 32.0 J cm^−3^ and Δ*f*(16F‐HDI‐A) > 31.0 J cm^−3^. The absence of prominent crystallization peak of 16F‐HDI on DSC suggests that a large driving force may be required for nucleation in fluorinated PU, although it may also mean that we are not able to observe a strong peak because the overall crystallinity is lower for this sample. A potential reason for this could be that the strong electron repulsion between fluorine atoms and the large atom size of the fluorine disrupt the nucleation process.^[^ ^54^, ^55^, ^56^ ^]^

We visualized the crystallization rate using polarized optical microscopy (POM) with a temperature‐controlled stage (Figure 5b). We first heated the sample to 200 °C to melt crystals, then cooled the sample to room temperature at a rate of 5 °C min^−1^. We began timing as soon as the first small crystalline region started to form. After 4 min, 16F‐HDI exhibited small crystal nucleation with less areal coverage compared to 15E‐HDI and 16H‐HDI, which displayed strong crystallization signal after just 1 min. To further explore this feature of fluorine, we compared the series of aromatic PUs using MDI and the same diol, named 16F‐MDI, 15E‐MDI, and 16H‐MDI. The POM images under crossed polarizers (90°) are shown in Figure 5c. The crystallization of 16F‐MDI is enhanced compared to 16F‐HDI, although it remains slower and weaker compared to 15E‐MDI and 16H‐MDI.

The slower process in spherulite growth is referred to as secondary crystallization.^[^ ^51^ ^]^ Although a larger driving force existed in the spherulite growth of fluorinated PU, the process remains slower than other nonfluorinated PUs. This phenomenon is potentially attributed to the lower crystallization temperature of the fluorinated PU and possible repulsive interactions between fluorine atoms.^[^ ^57^, ^58^ ^]^

To better understand the crystallization fraction in polyurethanes and commercial polymers, we derive the difference in molar entropy Δ*S* between the rubbery state and the semicrystalline state^[^ ^59^ ^]^ from the change in enthalpy measured by DSC (details of our procedure for calculating Δ*S* are described in Supporting Information and Figure S27.) We compare trends in Λ to Δ*S* in Figure 5d. Thermal conductivity increases with Δ*S*, suggesting that more ordered crystalline structures favor heat transfer. However, at the same level of Δ*S*, fluorinated polyurethanes generally exhibit lower Λ. This suggests that the low Λ of fluorinated polymers is not solely dependent on reduced crystallinity. Additional evidence is required to fully understand the underlying factors, leading us to the next step of measuring sound velocities.

### Lower Speed of Sound (*v*_l_) in Fluorinated Polymers

**Figure 6:**
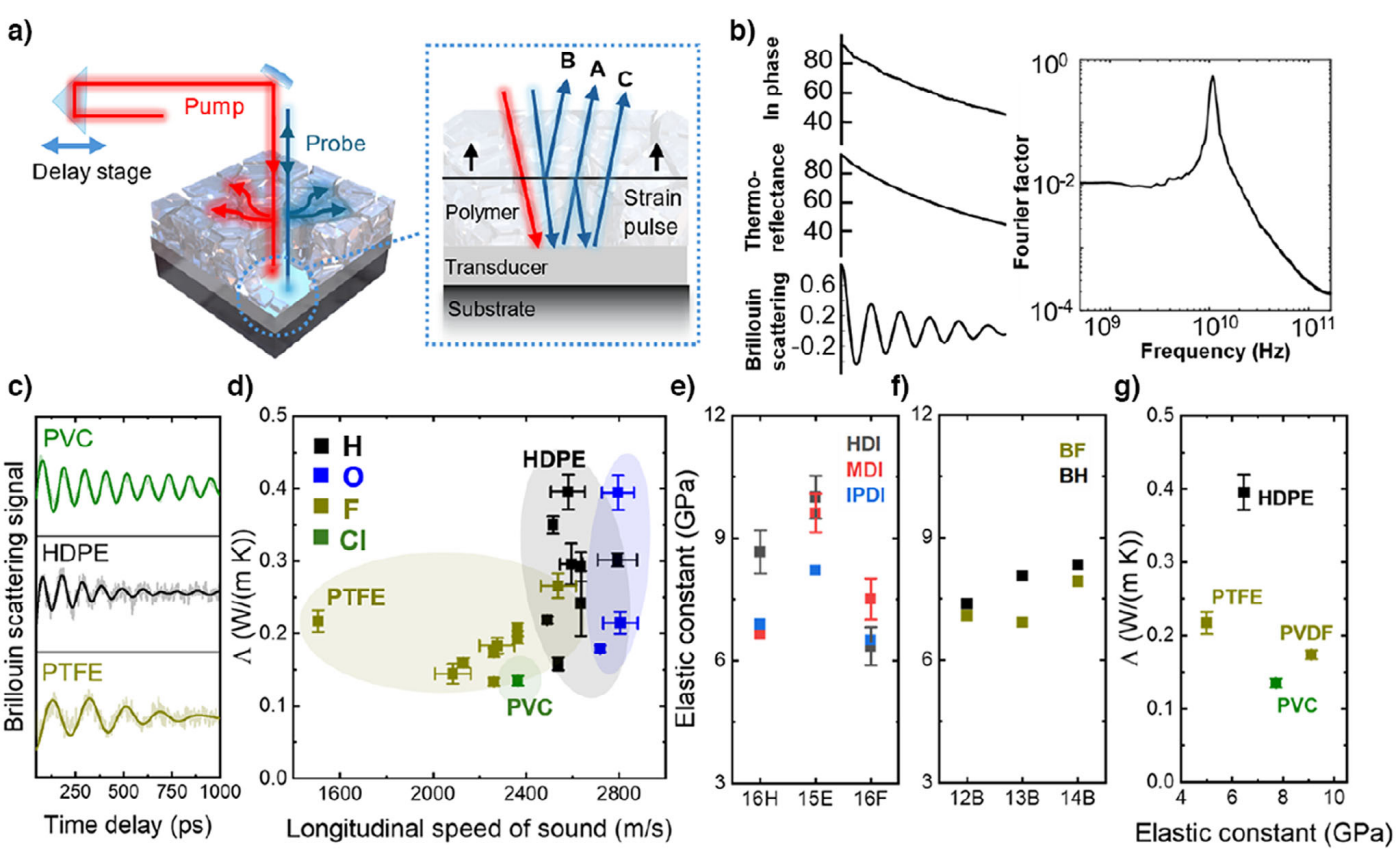
Brillouin scattering measured sound velocities in semitransparent samples. a) Illustration of Brillouin scattering measured in highly scattering polymer samples. The blue dashed line box highlights a microscopic view of the mechanism for interference generated by strain pulse. The scheme of interference mechanism was adapted from Ref. [60]. Short curved arrows pointing randomly denote the scattering of lasers within the crystallized samples. b) Working principle of extracting Brillouin scattering signal from the thermal signal, followed by the fast Fourier transform of extracted Brillouin scattering signal. c) Extracted Brillouin scattering signals of amorphous PVC and highly crystallized HDPE and PTFE samples. The light‐colored dots represent the extracted data, and dark‐colored lines represent the smoothed data, obtained by taking the mean over a ten‐element sliding window. d) Summary of longitudinal speed of sound and e)–g) summary of ***C***_11_ of synthetic and commercial polymer samples.

We tested our initial hypothesis that fluorinated polymer should exhibit a lower speed of sound, motivated by the low speed of sound observed in fluorinated liquids. As illustrated in Figure 6a, we utilized our TDTR setup to measure the forced Brillouin scattering signals^[^ ^60^ ^]^ (forced Brillouin scattering is also known as time‐domain Brillouin scattering or picosecond interferometry). The energy of the pump optical pulses is partially absorbed by the metal film transducer, generates a thermal stress, and launches longitudinal strain pulses that propagate in both forward and backward directions. The probe laser pulse that is reflected by the transducer (A) interferes with a weak reflection from the strain pulse (B) and a secondary weak reflection that involves a reflection from the strain pulse and two reflections from the transducer (C). The coherent interference of beams A, B, and C, depends on the distance travelled by the strain pulse where C only creates small perturbation on A. This interference modulates the intensity of the reflected probe and is detected by a photodiode and lock‐in amplifier synchronized to the modulation frequency of the pump beam. The period of this interference signal is related to the longitudinal speed of sound by $v_{l}=\frac{\lambda }{2n\tau }$, where *λ* is the wavelength of probe laser, *n* is the refractive index of sample, and *τ* is the period of the interference.

Forced Brillouin scattering typically requires highly transparent samples to obtain clear interference signals. However, most of the polymers in this study are semicrystalline and spatial inhomogeneities in the optical index of refraction cause significant scattering of the pump and probe lasers, resulting in interference signals that are difficult to distinguish from noise. As shown in Figure 6b, the in‐phase signal consists of a thermal reflectance signal from the metal film transducer combined with weak Brillouin scattering signals. This example is for 16H‐HDI, which exhibits the highest crystallinity in this study. We decomposed the in‐phase signal to extract the long‐term thermal reflectance signal, allowing for the analysis of periodic weak Brillouin scattering signal within a thermoreflectance signal that has 300 times higher amplitude.^[^ ^61^, ^62^ ^]^ By applying a fast Fourier transform to the extracted Brillouin scattering signal, we identified a clear frequency peak, which was then used to calculate the longitudinal speed of sound for the highly scattering crystalline samples. In our approach, the frequency resolution of the fast Fourier transforms is limited to 5%, and therefore the resolution of speed of sound measurement is also 5%. A method for measuring forced Brillouin scatting signals in strongly scattered liquids was described by Maznev et al., but in the geometry used in their study, the pump and probe beams do not pass through the sample.^[^ ^63^ ^]^

Figure 6c displays representative Brillouin scattering signals of standard polymers. PVC is amorphous and demonstrates clear Brillouin oscillations with a slow decay of amplitude. HDPE and PTFE are semicrystalline and exhibit noisier signals that decay within 800 ps. The calculated longitudinal speeds of sound for PVC, HDPE, and PTFE are 2.36, 2.58, and 1.51 km s^−1^, respectively, showing a significant decrease in speed when the polymer is fully fluorinated.

Brillouin scattering characterizes the speed of sound at frequencies on the order of 10 GHz.^[^ ^64^ ^]^ This high frequency affects the elastic properties of the polymers, making these speeds of sound unique at this frequency. However, the measured values for standard polymers are close to those characterized at lower frequencies as most sound velocity measurements are done at around 10 MHz using quartz transducers.^[^ ^65^, ^66^, ^67^ ^]^ We summarized the measured speeds of sounds in Figure 6d. As indicated by the light‐yellow circle, fluorinated polymers consistently exhibited smaller longitudinal speeds of sound. Fluorinated polymers generally also have smaller refractive indexes. For example, for aliphatic fluorinated PUs, the refractive index *n* = 1.44 of PVDF was used in the calculations.^[^ ^68^ ^]^ For black‐circled aliphatic PUs, the refractive index *n* = 1.51 of HDPE was applied, and for blue‐circled aliphatic PUs, that of polyethylene glycol *n* = 1.48 was used.^[^ ^69^ ^]^

We observed a reduced speed of sound in fluorinated polymers, particularly in PTFE, which exhibits an exceptionally low sound velocity of 1.50 km s^−1^. From our observations of increased average molecular distances and slower crystallization dynamics in fluorinated polymers, we conclude that the strong electronegativity of fluorine weakens the interactions between polymer chains. Furthermore, fluorine is heavier than hydrogen, and this added density results in a reduced speed of sound.

In our previous study, a strong correlation was observed between Λ and the longitudinal modulus (*C*_11_): larger moduli are correlated with larger *Ʌ*.^[^ ^44^, ^70^ ^]^ However, this correlation was not evident in our study of partially fluorinated polymers. The *C*_11_ were calculated using the density measured by pycnometry and the speed of sound measured by Brillouin scattering, *C*_11_ = $\rho v_{l}^{2}$ (Figure 6e,f). Crystallized PUs are expected to exhibit higher *C*_11_ and higher thermal conductivities to the amorphous PUs.^[^ ^71^ ^]^ However, this tendency is not clearly observed (Figure S28). PVDF is a counter‐example, exhibiting high crystallinity and high *C*_11_, but low thermal conductivity (Figure 6g). Analysis by WAXS reveals that the average molecular distance of PVDF is approximately 0.48 nm, compared to 0.41 nm for PTFE (Figure S10). In conclusion, for crystallized samples, the thermal conductivity shows a weak relationship with the *C*_11_ and the average molecular distance and chemical structures of polymers should also be considered.

## Conclusion

Rationally manipulating the molecular structure of polymeric materials for enhanced thermal insulation has been rarely studied. Our work shows that the addition of fluorine to polymers reduces Λ due to decreased crystallinity, lower atomic density, and lower speed of sound. In this study, the lowest Λ we observed was 0.13 W m^−1^K^−1^ at room temperature for the aliphatic fluorinated polyurethane16F‐IPDI. A thermal conductivity of 0.13 W m^−1^K^−1^ is equal to the lower limit of the thermal conductivity of conventional amorphous polymers, specifically amorphous PVC. These effects, stemming from the large atomic size and high electronegativity of fluorine, contribute to the microstructural differences observed in fluorinated polymers compared to their nonfluorinated counterparts. We observed that this phenomenon was strongly dependent on the chemical structures of the polymer backbone: fluorination decreases crystallinity of aliphatic polyurethane. However, for aromatic polyurethanes, the decrease in crystallinity created by fluorination is not significant. In this case, thermal conductivity is generally enhanced in comparison to aliphatic polyurethanes due to increased elastic constant from aromatic structures (Figure S28). The improved analysis of forced Brillouin scattering enables accurate measurement of the speed of sound in highly scattered samples, thereby extending the applicability of this method.

This study provides a rich database of structure–property relationships that will help guide future work in designing polymers with low intrinsic Λ. Fluorinated polymers are widely utilized in industry for their outstanding hydrophobicity, chemical resistance, and heat resistance.^[^ ^72^ ^]^ Our study provides insights that fluorinated polymers have generally lower thermal conductivity, offering benefits for industry where thermal insulation is desired. Machine learning offers a promising approach for establishing design principles between Λ and chemical structures.^[^ ^73^, ^74^ ^]^ Most machine learning studies for predicting the relationships between chemical structures and thermal conductivity rely, however, predominantly on molecular dynamics (MD) simulations for training. Experimental data are necessary for validation. We anticipate that our experimental work will provide an important step in the validation of computational studies and advance the development of machine learning models for the thermal conductivity of polymers.

## Supporting information

Fluorinated liquid thermal conductivity, optics illustration of TDTR and D‐TOPS, TGA, ATR, and FT‐Raman spectra of PUs, representative fitting of WAXS data, comparison between DSC measured crystallinity and WAXS fitted crystallinity, odd‐even effects in PUs, SAXS of amorphous PUs, all orientation SAXS and WAXS of PUs, sensitivity analysis of TDTR and TOPS, in‐plane and through‐plane thermal conductivity of PUs, DSC spectra of PUs and conventional polymers, elastic constant of PUs and conventional polymers, synthesis details and NMR of 12BF and 13BF.

## Author Contributions

The manuscript was written through contributions of all authors.

## Conflict of Interests

The authors declare no conflict of interest.

## Supporting information



Supporting Information

## Data Availability

The data that support the findings of this study are available in the Supporting Information of this article.
