# Supplementary material for: Reduction of the Thermal Conductivity of Polyurethanes by Fluorination: Impact of Crystallinity, Atomic Density, and Sound Velocity
Source: Angew Chem Int Ed Engl. 2025 Apr 25;64(25):e202503497. doi: 10.1002/anie.202503497 (PMC12171350; doi:10.1002/anie.202503497)
Supplement: Supplementary file 1 — Supporting Information [file ANIE-64-e202503497-s001.pdf]

Supporting information

for

Reduction of the Thermal Conductivity of Polyurethanes by Fluorination: Impact of Crystallinity, Atomic Density, and Sound Velocity

Author list: Jingyi Zhou [1,2,4], Chen Chen [1,2,4], Jinchi Sun[1,4], Thomas R. Fielitz[5], Weijun Zhou[6], David G. Cahill [*1,4], Paul V. Braun [*1,2,3,4]

[1]Department of Materials Science and Engineering, Grainger College of Engineering, University of Illinois Urbana−Champaign, Urbana, IL, 61801, United States

[2]Beckman Institute for Advanced Science and Technology, University of Illinois Urbana−Champaign, Urbana, IL, 61801, United States

[3]Department of Chemistry, University of Illinois Urbana−Champaign, Urbana, IL, 61801, United States

[4]Materials Research Laboratory, University of Illinois Urbana−Champaign, Urbana, IL, 61801, United States

[5]The Dow Chemical Company, Midland, MI, 48667, United States

[6]The Dow Chemical Company, Lake Jackson, TX, 77566, United States

[*]Corresponding author email: d-cahill@illinois.edu, pbraun@illinois.edu

Instruments and Materials

1,6-Hexanediol (99%), diethylene glycol (99%), 2,2,3,3,4,4,5,5-Octafluoro-1,6-hexanediol (98%), isophorone diisocyanate (IPDI, mixture of cis- and trans- isomers), hexamethylene diisocyanate (≥ 99%), 4,4′-methylene bis (phenyl isocyanate) (98%), Dabco-33LV, Poly(tetrafluoroethylene) (powder, 200 µm particle size), Poly(vinylidene fluoride) (average Mw ~180,000 g/mole by GPC, average Mn ~71,000 g/mole), polyethylene (High density, Mn ~ 125,000 g/mole), polyvinyl chloride (average Mw ~43,000 g/mole, average Mn ~22,000 g/mole) were purchased from Sigma-Aldrich (St. Louis, MO). The synthesis of 12BF and 13BF diols were provided in supporting information, which yield as white solids. Quartz capillaries (1.5 mm, thin wall: 10 µm) were purchased from Hampton Research.

Thermo Nicolet iS50 FTIR was used to collect attenuated total reflectance spectrum (ATR) and FT-Raman spectrum by a 1064 nm excitation laser to ascertain the completion of the reaction and solvent removal. Fisher brand Isotemp Model 281A Vacuum Oven was used for PU curing. Discovery 2500 Differential Scanning Calorimeter was used for analyzing the glass transition temperature and crystallinity of bulk PU. X-Ray Scattering System comprised of a Xenocs GeniX3D Cu Kα X-ray source (1.54 Å) and a Pilatus 300 Detector was used to collect both small angle X-ray scattering (SAXS) and wide-angle X-ray scattering (WAXS). Q50 Thermogravimetric Analysis (TGA) was used to measure the mass loss of polymer samples across the temperature from room temperature to 600 °C. INSTEC mK2000 temperature controller equipped with an HCS421V stage was used to control the temperature during temperature dependent measurements of thermal conductivity. Pfeiffer HiCube turbo pump was used to generate vacuum for temperature dependent measurements. Gel permeation chromatography (GPC) was performed using a GPC equipped with an isocratic pump (1260 Infinity II, Agilent), and a differential refractometer detector (Optilab T-rEX, Wyatt Technology). Three size-exclusion columns (PLgel MIXED-B columns, 10 µm, 7.5 x 300 mm, Agilent) were connected and separations were performed at 40 °C using DMF containing 0.1 M LiBr, at a flow rate of 0.7 mL/min. Polyurethane samples were dissolved in 0.1 M LiBr DMF at a concentration of 3-5 mg/mL and filtered using a 0.45 µm PTFE filter before measurement.

The Λ was measured by both time-domain thermoreflectance (TDTR) and displacement thermo-optic phase spectroscopy (D-TOPS). The TDTR setup uses a Ti:sapphire laser to generate femtosecond pulses at 76.9 MHz repetition rate. The laser is split into pump and probe beams, where the pump beam passes through a mechanical delay stage to adjust the arrival times of the pump and probe pulses at the sample surface. The reflected probe

beam with a 1/e^2 beam size of 4.9 μm measured the changes in reflectivity of sample transducer. The pump beam is modulated by a 9.3 MHz square wave and the thermoreflectance signal of the probe is measured by a lock-in amplifier. We fit the ratio signal $r(t) = -V_{in}/V_{out}$ of the in-phase and out-of-phase components to a multilayer thermal transport model in the delay time range of 100 ps to 3600 ps.[1] The optics layout is given in **Figure S2**.

D-TOPS uses two superluminescent diodes (SLDs) that have wavelengths at 670 nm as probe beam and 780 nm as pump beam. The pump and probe beams have an offset along the vertical direction on the sample surface. The offset is generated by a motorized actuator and precision gimbal mount. A quadrant-cell photoreceiver connected to a lock-in amplifier collects the probe beam deflection signal that are synchronous with the modulation of the pump beam. We fit the in-phase and out-of-phase signal in the range from 30 Hz to 30 kHz with a heat conduction and thermoelastic deformation model to get the thermal conductivity and coefficient of thermal expansion.[2] The optical layout of the D-TOPS system is illustrated in **Figure S3**.

Polyurethane (PU) synthesis

Diols, isocyanates, and catalysts are stored in boxes with a dry nitrogen purge to suppress contamination by water vapor. Prior to use, diols and isocyanates were dried at 50 °C under vacuum for 24 hours. Inadequate removal of moisture can result in reactions between isocyanate and water, generating urea as described in the supplement (**Figure S4**). Aliphatic diols (**Figure 1b)** were first heated to 80 °C in a 20 mL vial with continuous stirring at 400 rpm, followed by an addition of 1 wt.% Dabco-33LV and mixed for 10 min. Isocyanates (**Figure 1c**) were mixed at a 1:1 molar ratio of isocyanate to diol to initiate for bulk condensation at 80 °C. After 24 hours, the formed PU was further cured at 120 °C under vacuum for 16 hours.

Anhydrous DMSO was used to dilute aromatic diols due to their high reactivity. The isocyanates were added to the diluted aromatic diols at a 1:1 molar ratio. The reaction mixture had a final concentration of 100 mg/mL. After stirring the reaction mixture continuously at 400 rpm at 80 °C for 24 hours, pre-PUs precipitated as white solids. The DMSO was removed under reduced pressure at 80 °C, and the resulting residue was further cured at 120 °C for 16 hours. After curing, the DMSO is further removed by dialysis of PUs in acetone three times in 50 mL centrifuge tubes. Aliphatic diols with aromatic isocyanate follow the same protocol as using aromatic diols.

Preparation of samples for thermal measurements

After synthesis, we prepared PU samples for thermal measurements in two ways. For PUs prepared by bulk condensation, we melted the polymer on top of a polished fused silica substrate at 200 °C under vacuum (~1x10^{-2} Pa) in a small well created by a 200 μm

thick metal washer, and subsequently cooled it down to room temperature at a rate of 5 °C/min using a temperature controlled hot plate. After reaching room temperature, the sample was immersed into liquid nitrogen and peeled off using a razor blade to produce a smooth surface. For PUs prepared by solution condensation, the PU was positioned between two polished fused silica substrates and hot-pressed under a weight of 1 ton at 150 °C over a circular 1.8 cm^2 area (54 MPa). After cooling at the same rate of 5 °C/min, one of two silica substrates was peeled off using razor blade to produce a smooth surface. The formed surface was coated with ~3 nm Nb-V followed by ~80 nm Al using magnetron sputtering to serve as a transducer for D-TOPS and TDTR measurements. The thin layer of Nb-V is intended to block the reaction of Al with fluorinated polymers, which has been observed in magnetron sputtering of Al/PTFE.[3]

We included standard polymers such as high-density polyethylene (HDPE), polyvinylidene fluoride (PVDF), polytetrafluoroethylene (PTFE), and polyvinyl chloride (PVC) to validate the accuracy of Λ measurements and to compare their microstructures and thermophysical properties with synthesized PU. All standard polymer samples were slow cooled at the same rate of 5 °C /min. The starting temperature was selected based on their respective degradation and melting temperatures.[4] For example, the starting temperature for PTFE was chosen at 375 °C, above the melting temperature and below its degradation temperature of 400 °C.

Preparation of sample for Brillouin scattering

Samples for forced Brillouin scattering were prepared by slow cooling polymers at a rate of 5 °C/min on top of Al (~80 nm)/silica substrate (500 μm). An additional layer of glass cover slide (~100 μm) was placed on top of the polymer, and a 1 kg weight was used to press the polymer, ensuring better contact with the substrate. The typical thickness of the polymer layer was approximately 50 μm, as measured by a micrometer.

Supplemented figures

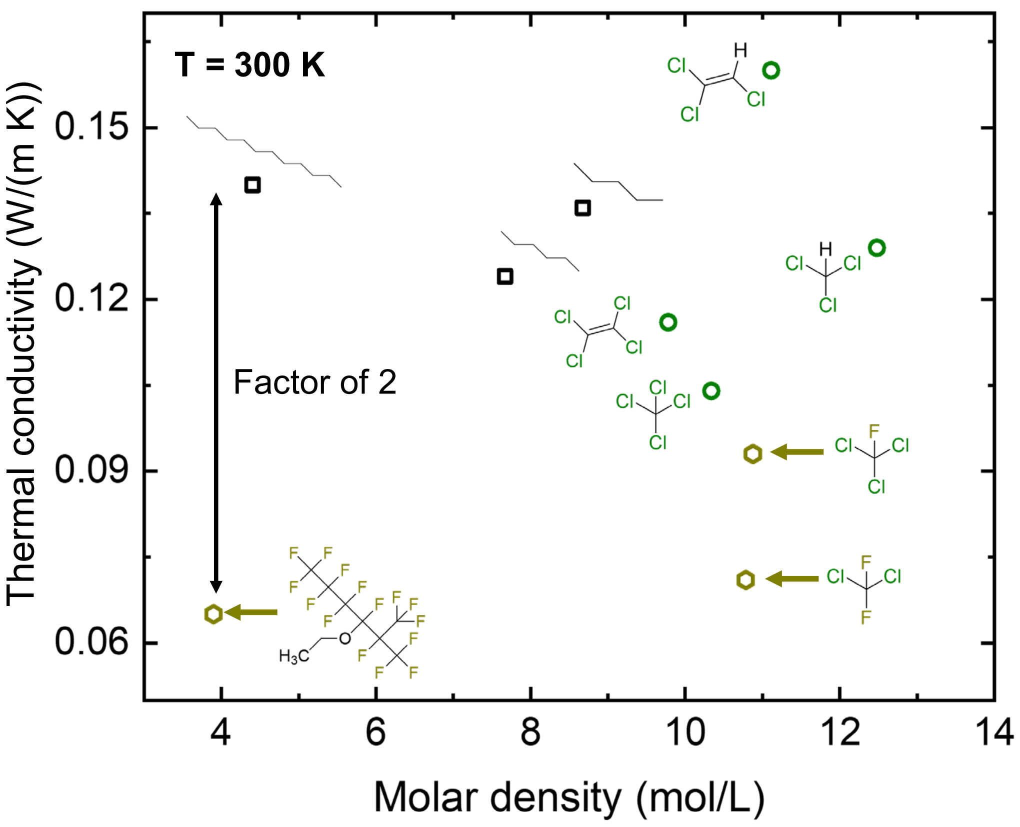

Figure S1. Thermal conductivity of similar fluorinated, chlorinated, and non-halogenated liquids at 300 K. The highly fluorinated structure at approximately 4 mol/L is 3M Novec 7500 (HFE 7500).

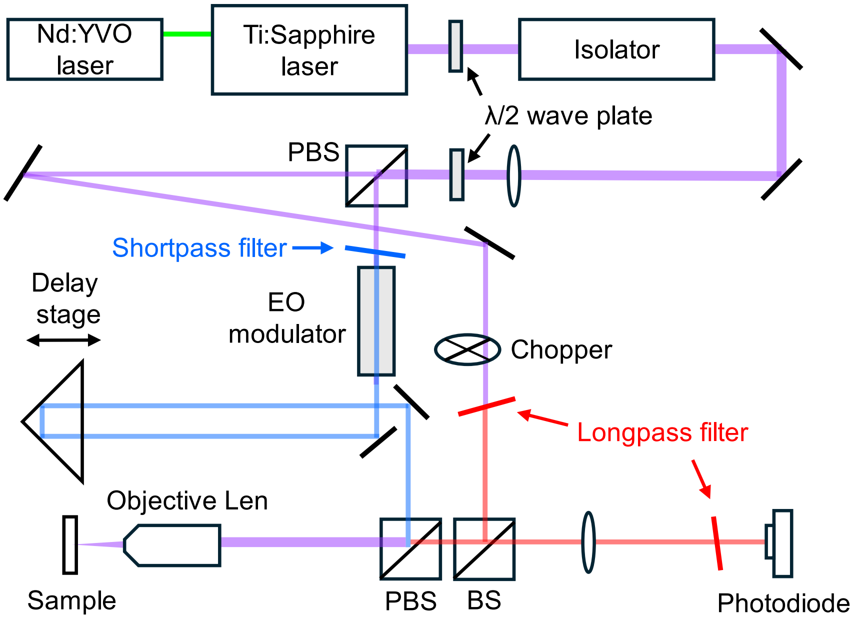

Figure S2. Optics setup for time-domain thermoreflectance (TDTR): The purple line represents the overlapping pump and probe laser, the blue line represents the pump laser, and the red line represents the probe laser. A camera (not depicted) is also incorporated into the setup for focusing on the sample surface.

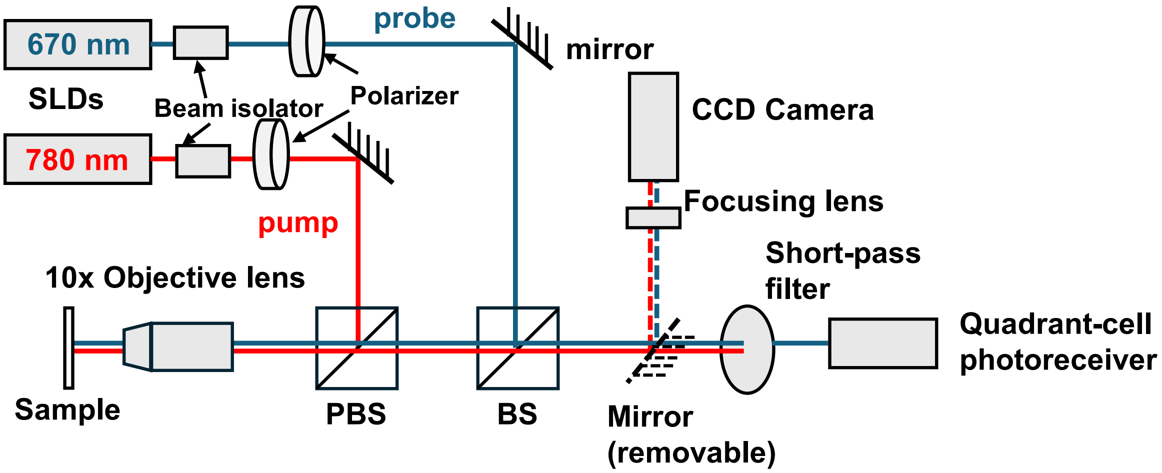

Figure S3. Simplified illustration of optics setup for displacement-thermo-optic phase spectroscopy (D-TOPS). Blue line represents the probe laser, and red line represents the pump laser. Polarizers are used for adjusting the laser power arriving at sample surface. SLDs are superluminescent diodes.

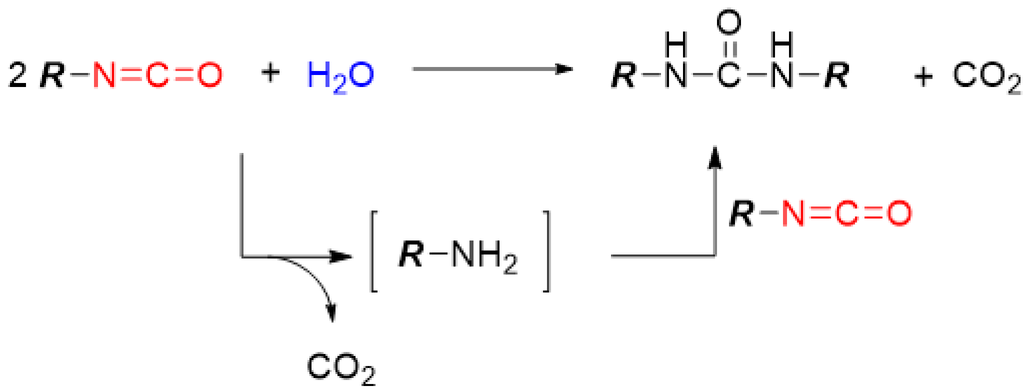

Figure S4. Illustration of potential side reactions between isocyanate and water contaminates.

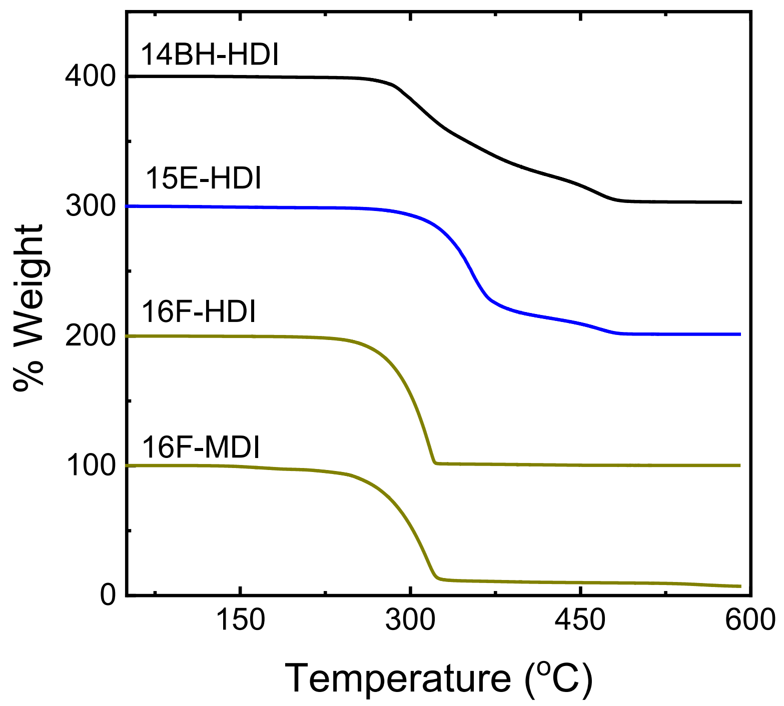

Figure S5. Thermogravimetric analysis (TGA) thermogram of representative PUs. 16F-HDI and 15E-HDI were synthesized via bulk polymerization; 14BH-HDI and 16F-MDI were synthesized through solution polymerization. The structures of monomers for synthesizing these PUs can be found in **Figure 1**. For example, 14BH-HDI is synthesized from diol labeled 14BH and isocyanate labeled HDI. Offset between each plot is 100.

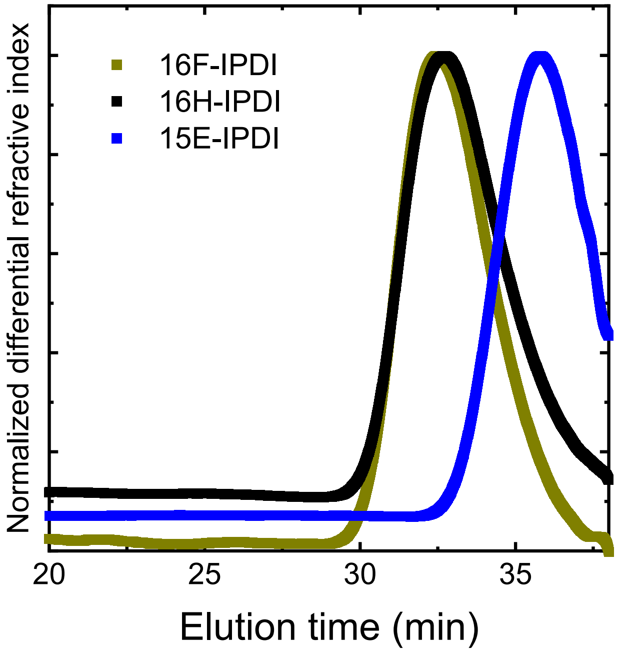

Figure S6. Gel permeation chromatography (GPC) data for polyurethanes 16F-IPDI, 16H-IPDI, and 15E-IPDI.

Table S1. GPC analysis results of 16F-IPDI, 16H-IPDI, and 15E-IPDI.

Polyurethane	Mn (g/mol)	Mw (g/mol)	PDI
16F-IPDI	$1.67x10^4$	$2.03x10^4$	1.22
16H-IPDI	$9.06x10^3$	$1.28x10^4$	1.41
15E-IPDI	$2.55x10^3$	$3.18x10^3$	1.24

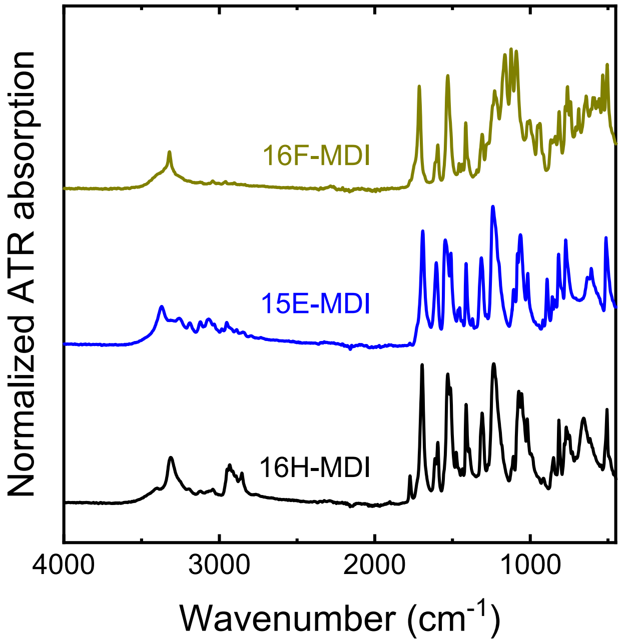

Figure S7. Attenuated total reflectance (ATR) spectra of MDI series polyurethanes synthesized via solution condensation.

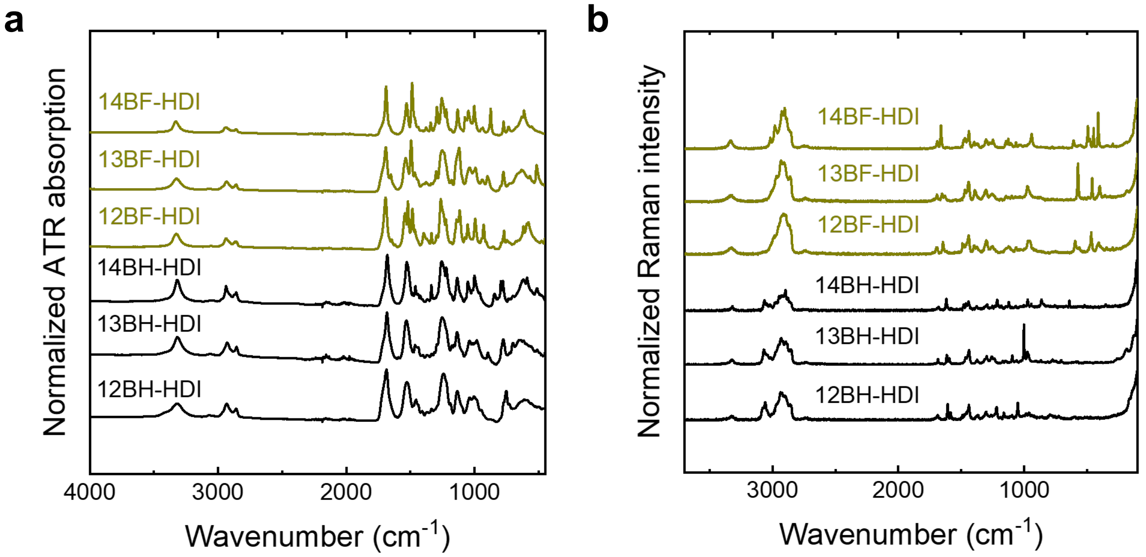

Figure S8. a) Attenuated total reflectance (ATR) and b) FT-Raman spectra of polyurethanes with fluorine substituted on aromatic ring or without fluorine on aromatic ring. The structural details of isocyanate and diols used for PUs can be found in **Figure 1**.

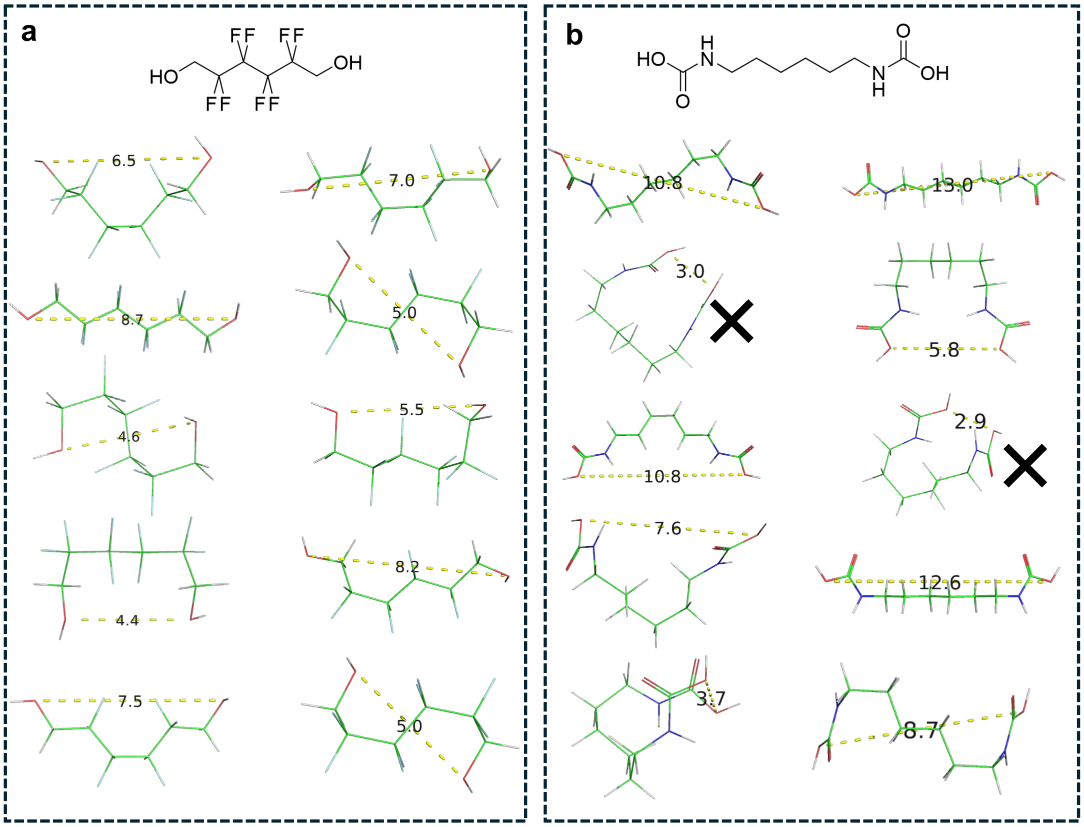

Figure S9. Estimation of the repeat unit length of PU 16F-HDI from the split structural components. a) 2,2,3,3,4,4,5,5-Octafluoro-1,6-hexanediol (16F).[5] b) hexamethylenedicarbamic acids were selected for the calculation in place of hexamethylene diisocyanate (HDI) to ensure inclusion of the carbonyl group.[6] The distances between oxygen atoms were measured in ten energy-favorable structures provided by PubChem using Pymol. To refine the estimation, two outlier conditions in hexamethylenedicarbamic acids (as black marked) were excluded, and the averaged distances for each molecule were calculated. For 16F, the average distance is 6.2 Å; for hexamethylenedicarbamic acids, the average distance is 9.1 Å. The estimation is based on addition of the two average distances, resulting in 1.5 nm, which aligns closely with the expected length scale measured by WAXS.

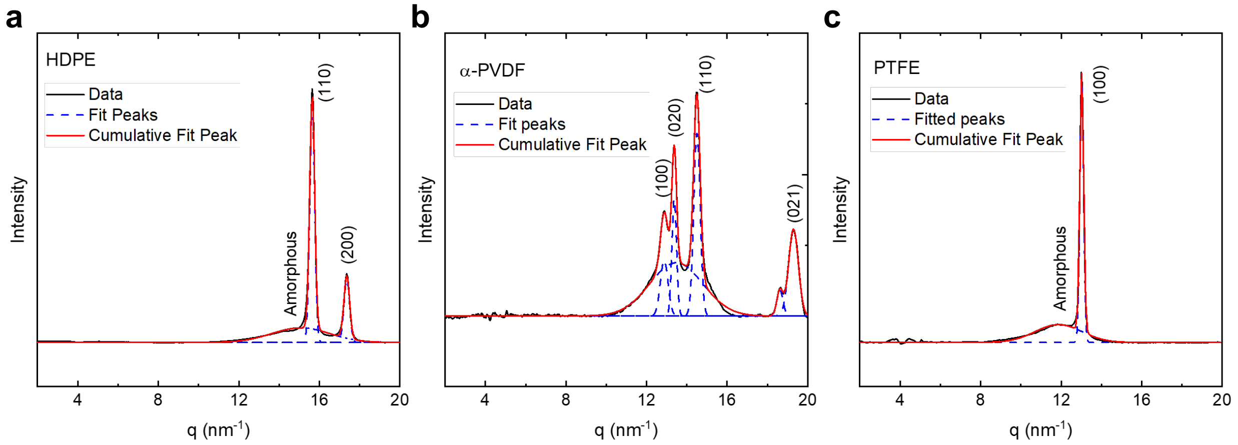

Figure S10. The Integration of wide-angle X-ray scattering (WAXS) peaks performed for a) HDPE: $X_{c,WAXS}$ = 61%, b) PVDF: $X_{c,WAXS}$ = 54%, and c) PTFE: $X_{c,WAXS}$ = 54%. Baseline correction was achieved through polynomial fitting (on the order of 4), and anchor points were snapped to measured data at q equals to 2, 4, 6, 8, 10, 20 nm^{-1}(for HDPE excluded 10 nm^{-1}). The black solid line indicates the WAXS signal, the blue dash line denotes the individually integrated peak, and the red solid line represents the cumulative integration.

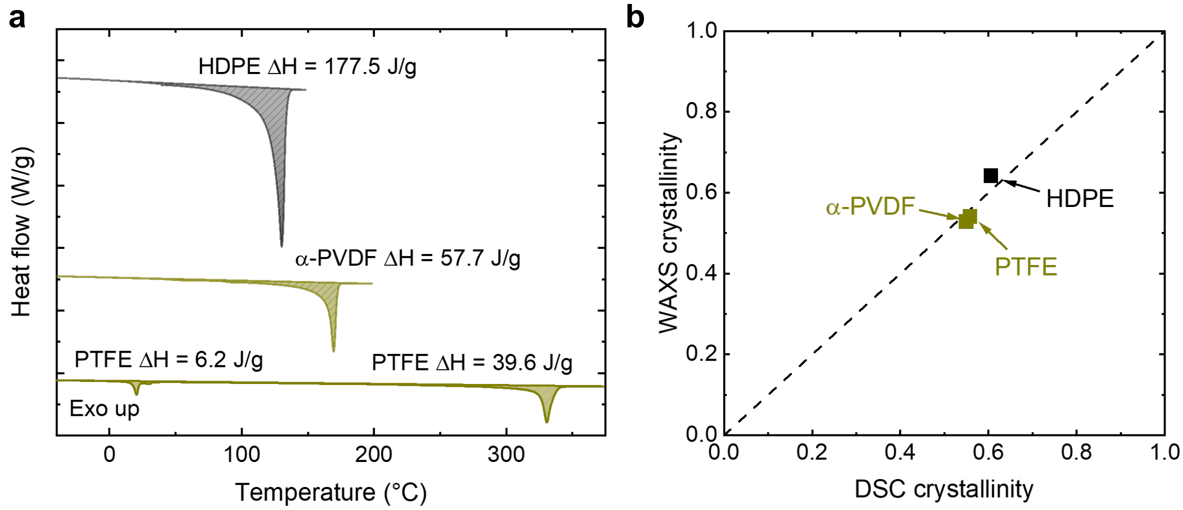

Figure S11. a) DSC enthalpy integration. The crystallinity derived from DSC was calculated using $X_{c,DSC} = \Delta H_{DSC}/\Delta H_{100\%\ crystallinity}$.The first peak of PTFE was added to consideration of crystallinity. The value of $\Delta H_{100\%\ crystallinity}$ was obtained from the literature.[7] b) comparison of crystallinity measurements between DSC and WAXS. The black dash line represents an equivalence that $X_{c,DSC} = X_{c,WAXS}$.

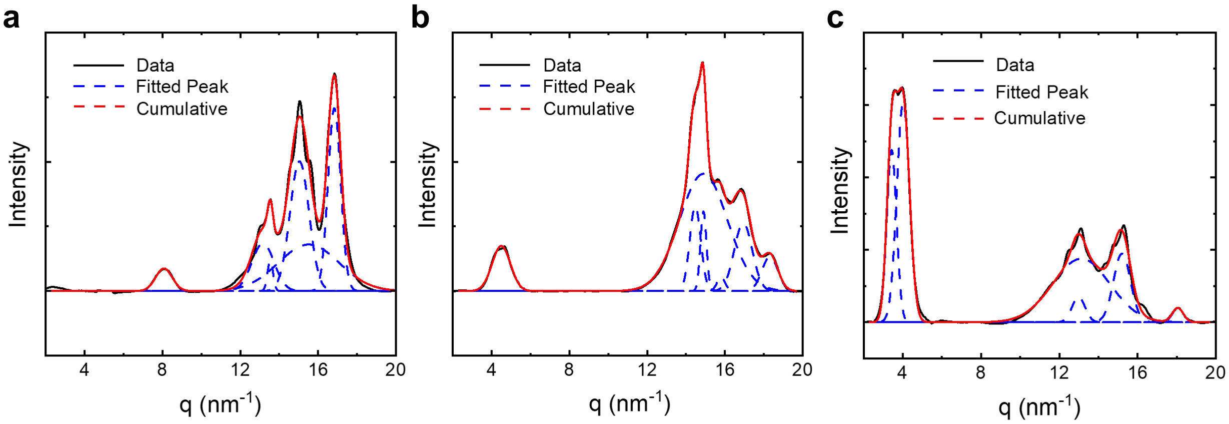

Figure S12. The integration of wide-angle X-ray scattering (WAXS) peaks for a) 16H-HDI: $X_{c,WAXS}$ = 68%, b) 15E-HDI: $X_{c,WAXS}$ = 37%, and c) 16F-HDI: $X_{c,WAXS}$ = 31%. Signal processing was conducted in accordance with the methodology described in **Figure S10**. For crystallinity measurements, only peaks in the q range of 10-20 nm^{-1} were considered. Peaks in range of 2-10 nm^{-1} were integrated to analyze the intensity of lamella packing.

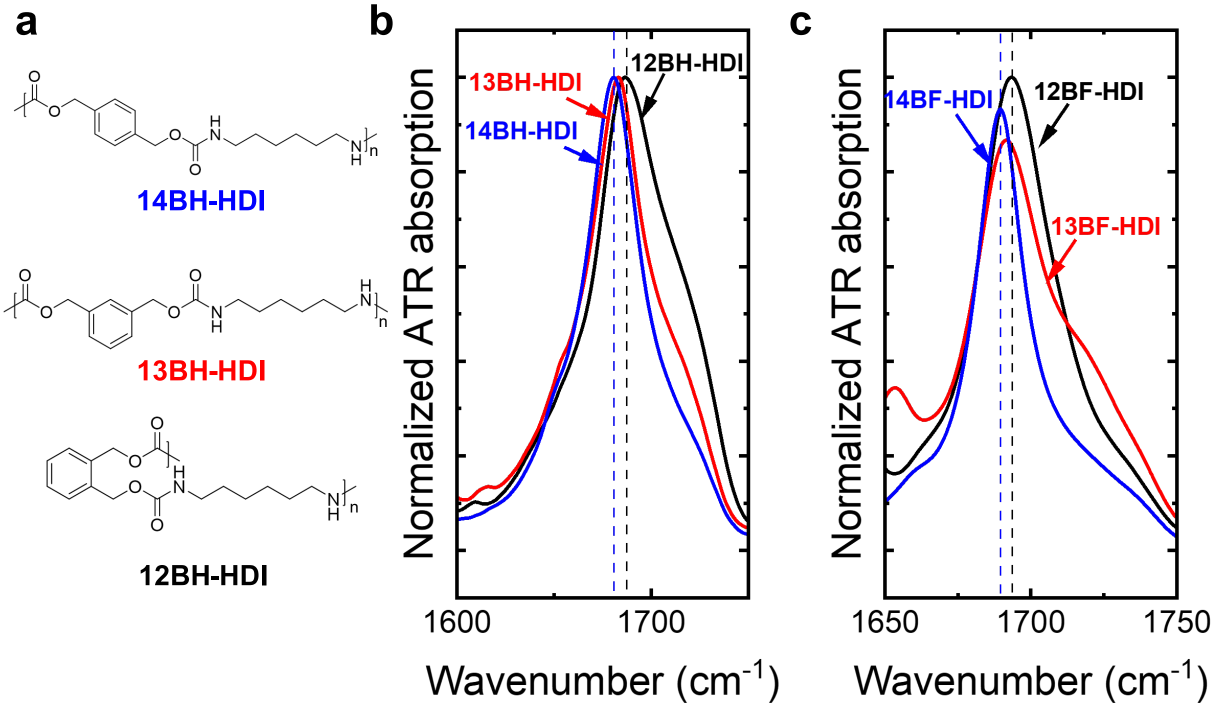

Figure S13. ATR carbonyl group wavenumber shifts in PUs synthesized from aromatic diols. a) Representative chemical structures of PUs compared in ATR spectra, where difference between BF and BH is that BF contains fully fluorinated aromatic ring. b)

Wavenumber shifts observed in the BH-HDI series of PUs. The blue dashed line indicates the carbonyl peak position for 14BH-HDI at 1681 cm^{-1}, and black dash line represents the carbonyl peak position for 12BH-HDI at 1688 cm^{-1}. c) The wavenumber shifts in BF-HDI PUs. The blue dash line corresponds to carbonyl peak position for 14BH-HDI at 1689 cm^{-1} and black dash line for 12BF-HDI at 1693 cm^{-1}.

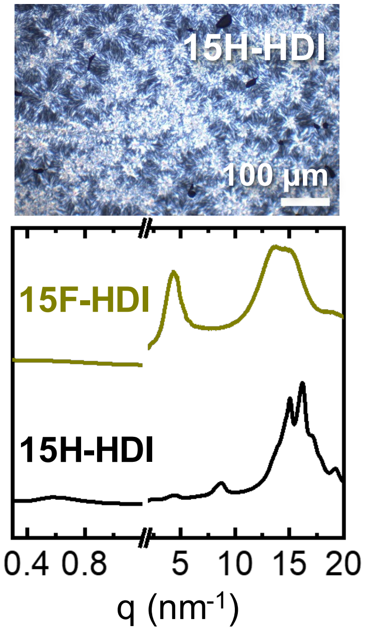

Figure S14. Polarized optical microscope (POM) images of 15H-HDI and combined X-ray scattering characterization of 15F-HDI and 15H-HDI for studying odd-even effects. Crystallization is only observed in 15H-HDI, where 15F-HDI exhibited no crystalline signal in POM.

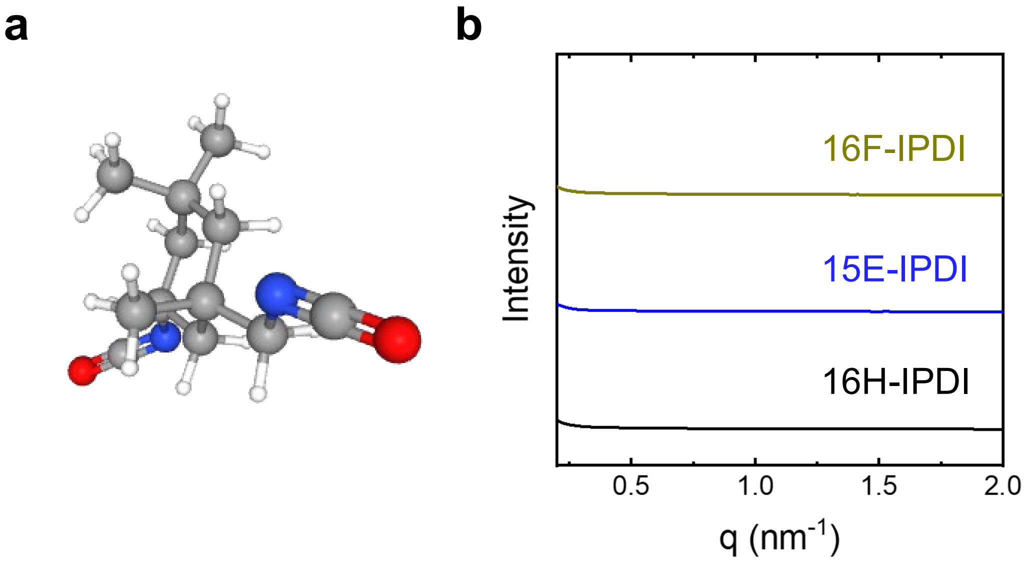

Figure S15. a) Steric structure of isophorone diisocyanate (IPDI), with atom colors as follows: grey for carbon, blue for nitrogen, red for oxygen, and white for hydrogen.[8] b) SAXS integration spectrum of 16F-IPDI, 15E-IPDI, and 16H-IPDI.

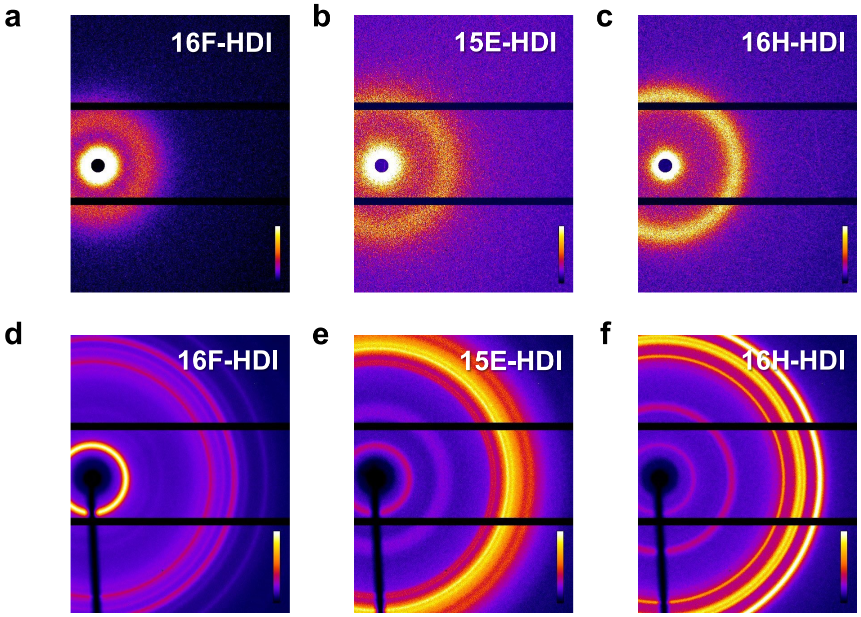

Figure S16. a), b), and c) Small angle X-ray scattering (SAXS) was employed to study the crystallinity of linear crystalized synthetic PUs across all angular orientations. The data of three samples were collected under the same exposure conditions. d), e), and f) Wide angle X-ray scattering (WAXS) of crystallized PUs. The scattering vector in these figures ranges from approximately 0.02 nm^{-1} to 2 nm^{-1} for SAXS and 2 nm^{-1} to 20 nm^{-1} for WAXS.

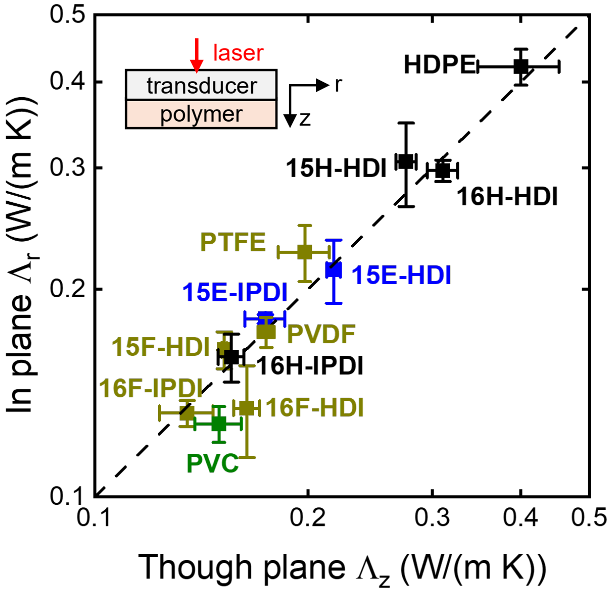

Figure S17. In-plane thermal conductivity measured by D-TOPS and through-plane thermal conductivity by TDTR. The black dash line represents the line of isotropy.

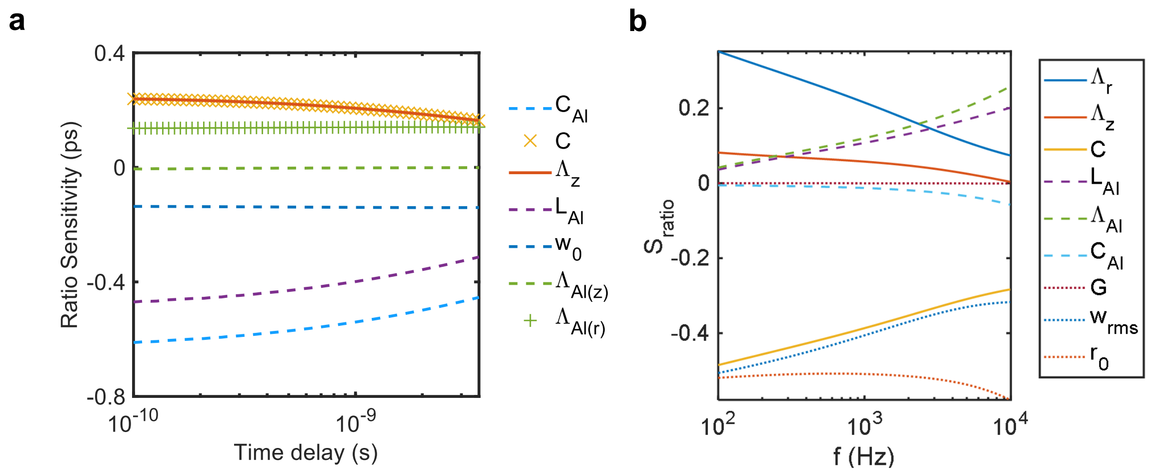

Figure S18. Sensitivity calculations using geometry of Al (80 nm)/polymer (infinite, thermally thick). a) For TDTR and b) for D-TOPS. The Nb-V layer was not separately included in the model since it is less than 3 nm thick and cannot be observed in picosecond acoustics data. The contribution of Nb-V layer was included in the thermal conductivity of Al layer, which was 110 W/(m K) measured by a four-point probe method. We assume each layer is isotropic and the parameters used in calculation are: Λ_{Al}: 110 W/(m K), L_{Al}: 80 nm, C_{Al}:2.44 MJ/(m^3 K), Λ_z or Λ_r (through-plane or in-plane thermal conductivity of polymer): 0.2 W/(m K), C (polymer heat capacity): 1.6 MJ/(m^3 K),

w_0(TDTR beam size): 4.9 μm, w_{rms} (TOPS beam size): 8.1 μm, r_0 (TOPS beam offset): 9.3 μm, $G_{Al/PU}$: 70 MW/(m^2 K).

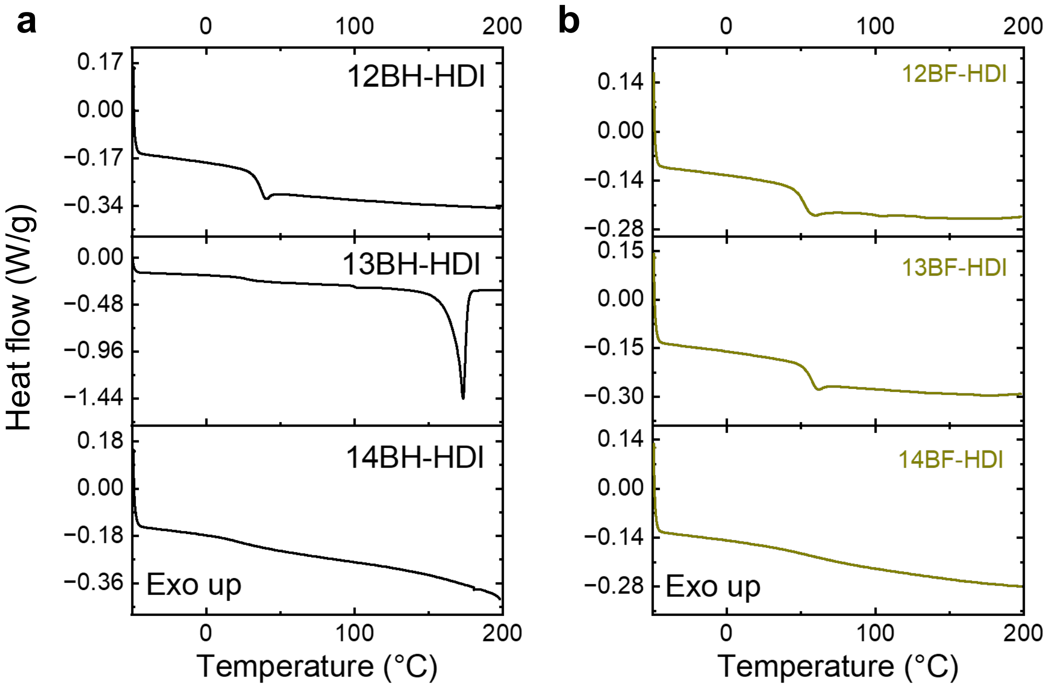

Figure S19. Differential scanning calorimetry (DSC) thermogram of a) BH-HDI and b) BF-HDI. Ramp rate: 10 °C/min.

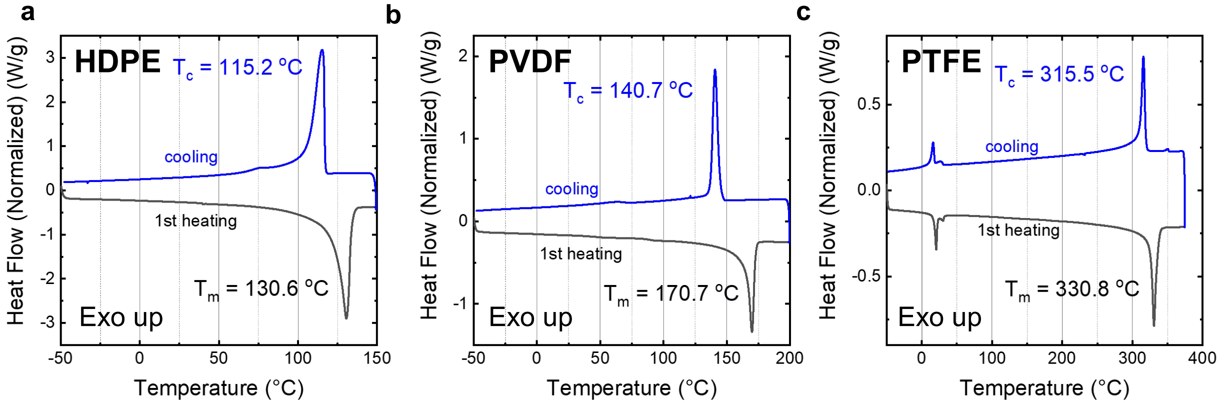

Figure S20. Differential scanning calorimetry (DSC) thermogram of standard polymers, including a) high density polyethylene (HDPE), polyvinylidene fluoride (PVDF), polytetrafluoroethylene (PTFE). The crystallization peak temperature (T_c) during the cooling process and the melting peak temperature (T_m) during the heating process are denoted. The temperature ramp rate is 10 °C /min for all samples.

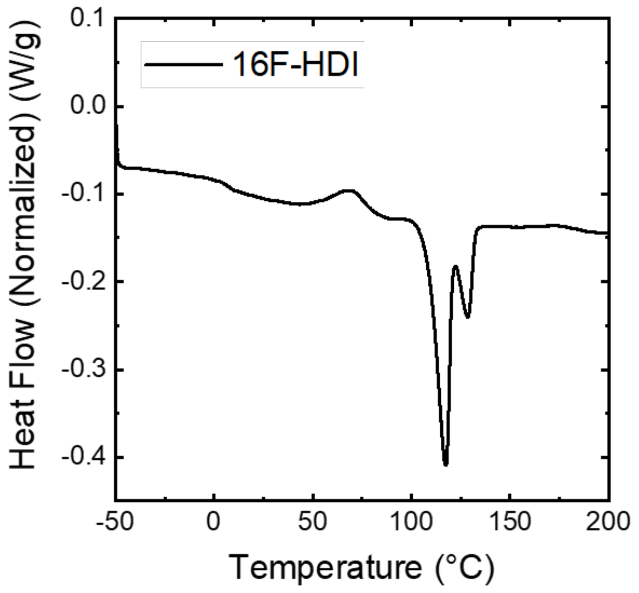

Figure S21. The DSC thermogram of 16F-HDI after slow cooling and holding overnight at room temperature before testing (10 K/min). This sample is the same sample as the one in **Figure 4c**, indicating that the crystallization is recoverable at room temperature.

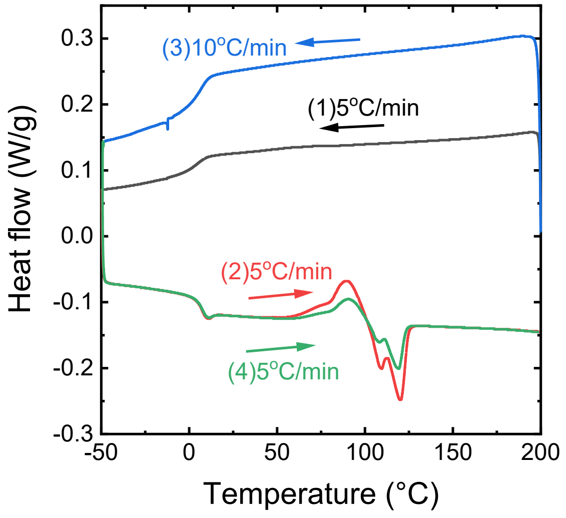

Figure S22. The DSC thermogram of 16F-HDI was analyzed using different cooling rates to observe the cold crystallization behavior. The process involved fast heating at 10 °C/min, followed by: 1) cooling at 5 °C/min (black line), 2) heating at 5 °C/min (red line), 3) cooling at 10 °C/min (blue line), 4) heating at 5 °C/min (green line). We observed that a slower cooling rate resulted in higher intensity of cold crystallization.

The integration of cold crystallization peak is consistent with the enthalpy integration of melting peak.

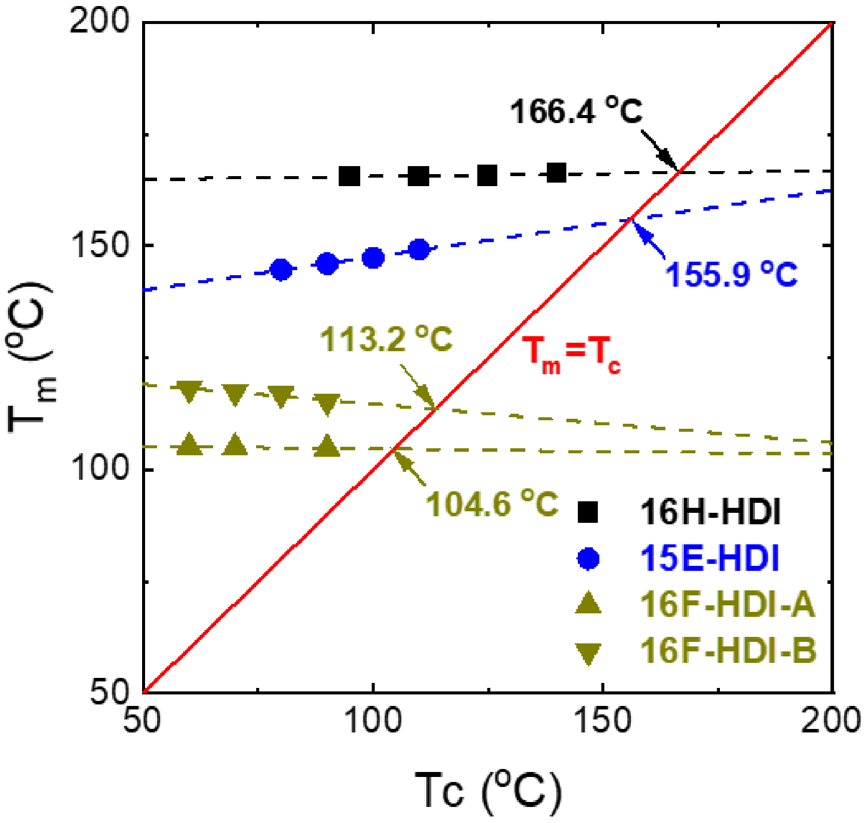

Figure S23. Hoffman-Weeks method measured equilibrium melting points of three PUs. For each PU, a linear regression line was fitted to the data points representing the relationship between the isothermal crystallization temperature (T_c) and the measured melting point (T_m) following crystallization. The equilibrium melting point for each PU was identified at the intersection of the fitted line with T_m=T_c line (red). 16F-HDI has two crystal phases defined as phase A and B.

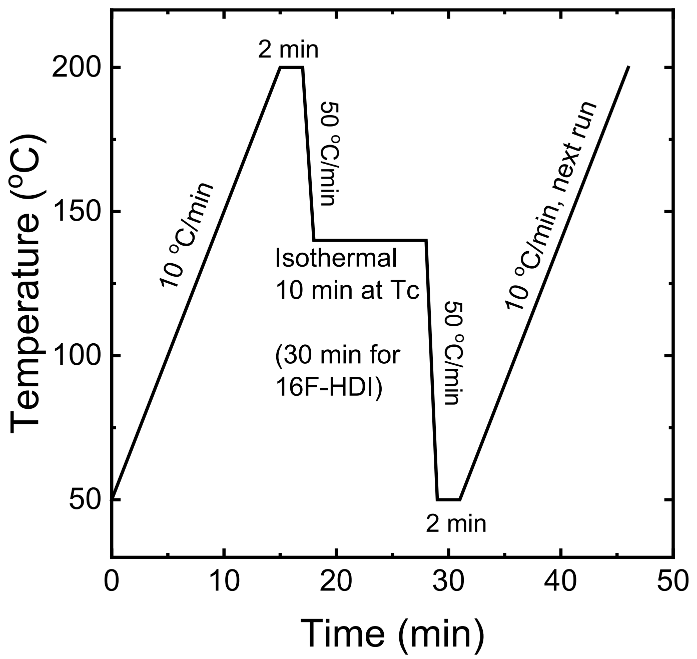

Figure S24. Representative DSC program settings for the isothermal crystallization cycle followed protocol described in Ref. [9]. Since 16F-HDI exhibits slower crystallization rate, the isothermal crystallization time was set to 30 min to ensure the crystallization occurred and could be reliably detected.

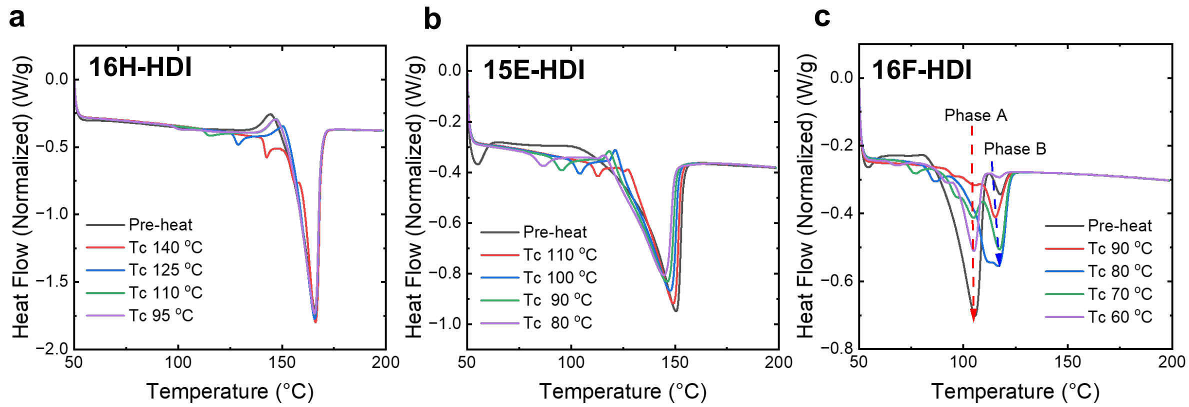

Figure S25. DSC thermograms for the melting processes of three PUs, after different crystallization temperatures. Notably, the 16F-HDI displayed multiple peaks during melting, with the observation that a lower crystallization temperature resulted in a more pronounced crystal peak at the main peak of pre-heat. We identified the peak indicated by the red arrow as crystal phase A, where the blue arrow represents phase B.

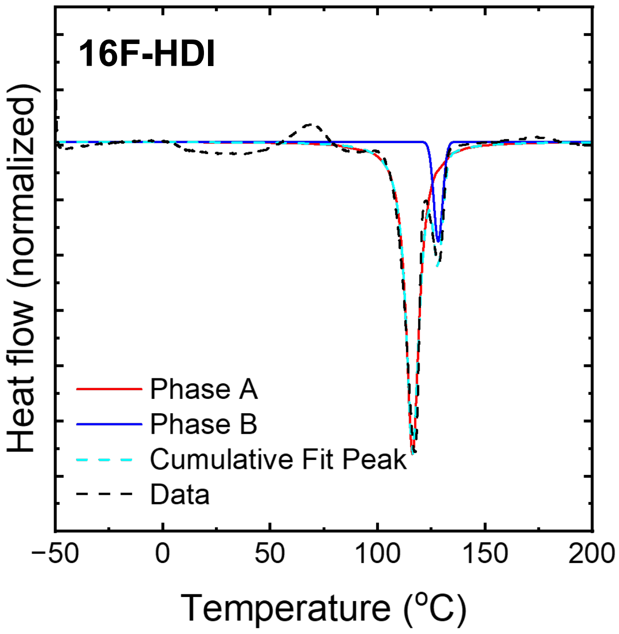

Figure S26. DSC thermogram fitting of 16F-HDI maintained at ambient conditions for 24 hours. The solid red and blue lines represent the fittings for two distinct crystalline phases A and B, respectively. Cyan dash line stands for cumulative fitting of A and B, and black dash line is experimental data. The fraction by integration for phase A is 87% and B is 13%. From WAXS shown in Figure S10c, 31% is the total crystalline fraction of polymer, where phase A contributes 87% and phase B contributes 13%.

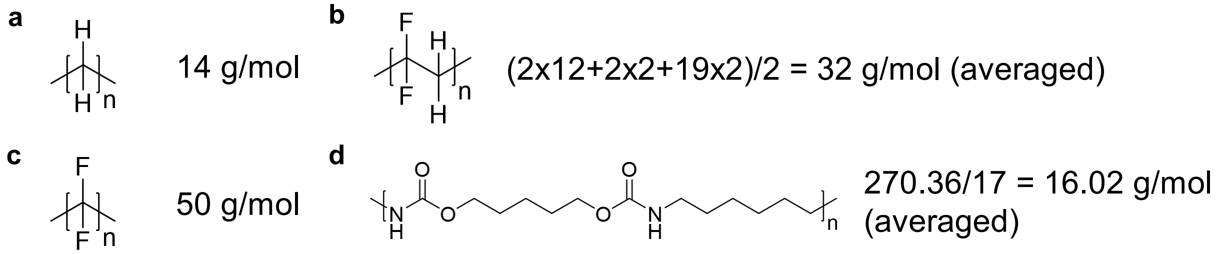

Figure S27. An example calculation of the average molecular weight of the backbone units: After averaging the molecular weight for each backbone atom to obtain M_{nor}, the entropy in units of R was calculated using the formula $\Delta S = \frac{\Delta H}{TR} M_{nor}$ (unitless), where R = 8.31 J/(mol-K), ΔH is the heat of fusion (unit of g/K), and T is the melting temperature.

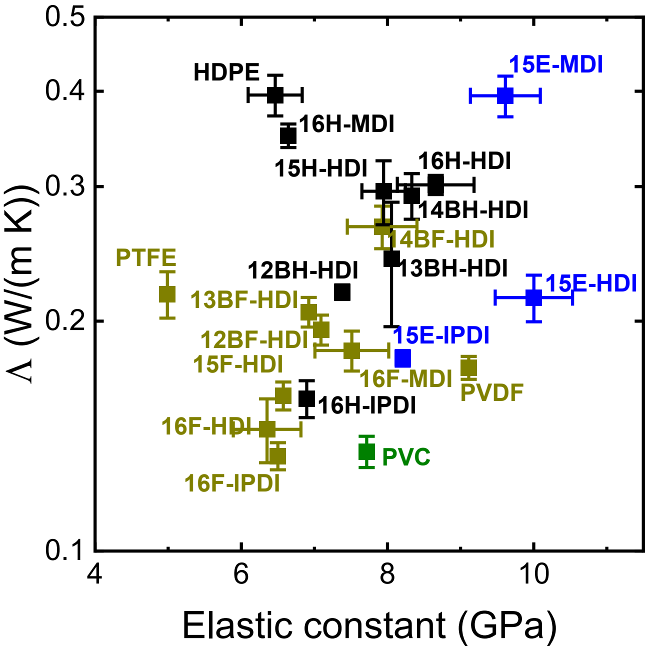

Figure S28. Summary of the longitudinal elastic constant C_{11} and thermal conductivity Λ. The polyurethanes labels including BF, BH, and MDI indicate they are aromatic polyurethanes.

Synthesis details

Materials used in synthesis and instruments

Lithium borohydride solution (2.0 M in THF), Borane tetrahydrofuran complex solution (1.0 M in THF) was purchased from Sigma-Aldrich. 2,4,5,6-Tetrafluorobenzene-1,3-dicarboxylic acid was purchased from Santa Cruz Biotechnology Inc. 3,4,5,6-Tetrafluorophthalic anhydride and 2,3,5,6-Tetrafluoro-1,4-benzenedimethanol were purchased from Oakwood Products Incorporated/Oakwood Chemical. 1H, 13C, 31P, 11B, 19F NMR spectra were collected on Carver B500 Bruker Avance III HD NMR Spectrometer equipped with a CryoProbe.

1) Synthesis of 12BF (*(perfluoro-1,2-phenylene)dimethanol*)

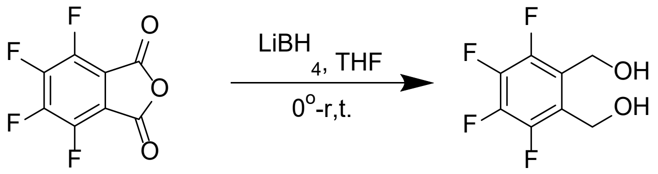

Figure S29. Synthetic route of *(perfluoro-1,2-phenylene)dimethanol*

The synthesis of 12BF followed the procedure from reference.[10] 4,5,6,7-tetrafluoroisobenzofuran-1,3-dione (1.10 g, 5.0 mmol, 1.0 equiv.), was added to a 100 mL round flask, followed by evacuated and refilled with dry nitrogen (x3). THF was pre-dried with 4 Å molecular sieves which has been activated under 240 ℃ for 8 hrs. After adding THF (30 mL) and reagent completely dissolved, the flask was cooled to 0 ℃ in an ice/water bath. LiBH4·THF solution (2M, 6.24 mL. 12.48 mmol, 2.5 equiv.) was added slowly into the flask with continuously stirring. The flask was then removed from the cold bath, and the reaction mixture was left stirring at room temperature for 16 hrs. The reaction was quenched by slowly adding 1M HCl followed by Millipore water. The obtained mixture was extracted with DCM (3x15 mL), followed by washing with brine (15 mL) and dried over Na_2SO_4, filtered via vacuum filtration, and concentrated using rotary evaporator. The crude product was purified via a silica gel column (EtOAc: Hex from 4:1 to 1:1) and dried under vacuum to yield white solid (yield: 60%).

^{1}H NMR (500 MHz, DMSO-d6): δ 5.36 (s, 2H), 4.63 (s, 4H).

^{13}C NMR (126 MHz, DMSO-d6): δ 146.58, 144.69, 140.23, 138.28, 125.24, 53.07.

^{19}F NMR (471 MHz, DMSO) δ -144.22, -158.13.

ESI$^-$ [$C_8H_6F_4O_2$-H^-] calculated 209.0, found 209.0.

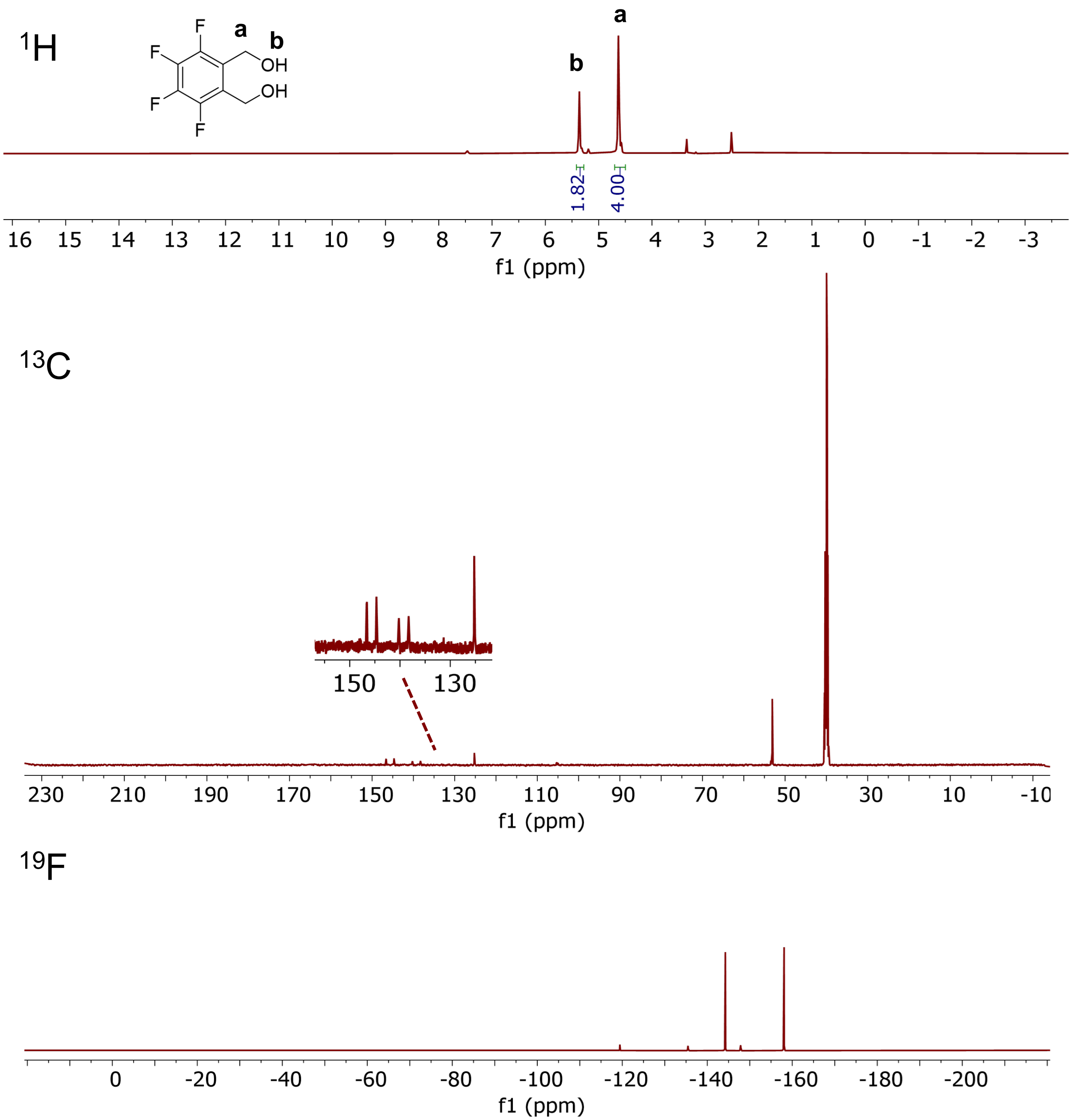

Figure S30. ^{1}H, ^{13}C, and ^{19}F NMR spectra of 12BF.

2) Synthesis of 13BF (*(perfluoro-1,3-phenylene) dimethanol*)

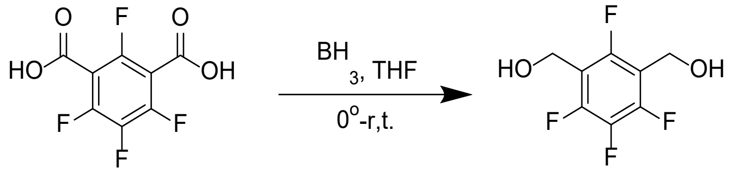

Figure S31. Synthetic route of *(perfluoro-1,3-phenylene) dimethanol*

The synthesis of 13BF followed the same procedure as 12BF except the reagents are different. 2,4,5,6-tetrafluoroisophthalic acid (0.49 g, 2.1 mmol,1.0 equiv.), was added to a 100 mL round flask, followed by evacuated and refilled with dry nitrogen (x3). 10 mL anhydrous THF was added to dissolve the reagent. BH3·THF solution (1M, 6.17 mL, 6.17 mmol, 3.0 equiv.) was added after the flask was cooled to 0 ℃ in an ice/water bath. After extraction and drying, crude product was purified by silica gel column by EtOAc: Hex from 4:1 to 1:1. White solid yielded after drying under vacuum (yield: 49%).

^{1}H NMR (499 MHz, DMSO-d_6) δ 5.42 (s, 2H), 4.49 (s, 4H).

^{13}C NMR (126 MHz, DMSO-d_6) δ 155.45, 153.51, 149.87, 147.90, 137.55, 135.61, 115.24, 51.15.

^{19}F NMR (471 MHz, DMSO-d_6) δ -124.76, -139.24, -166.41.

ESI$^+$ [$C_8H_6F_4O_2$+Na^+] calculated 231.0, found 231.0.

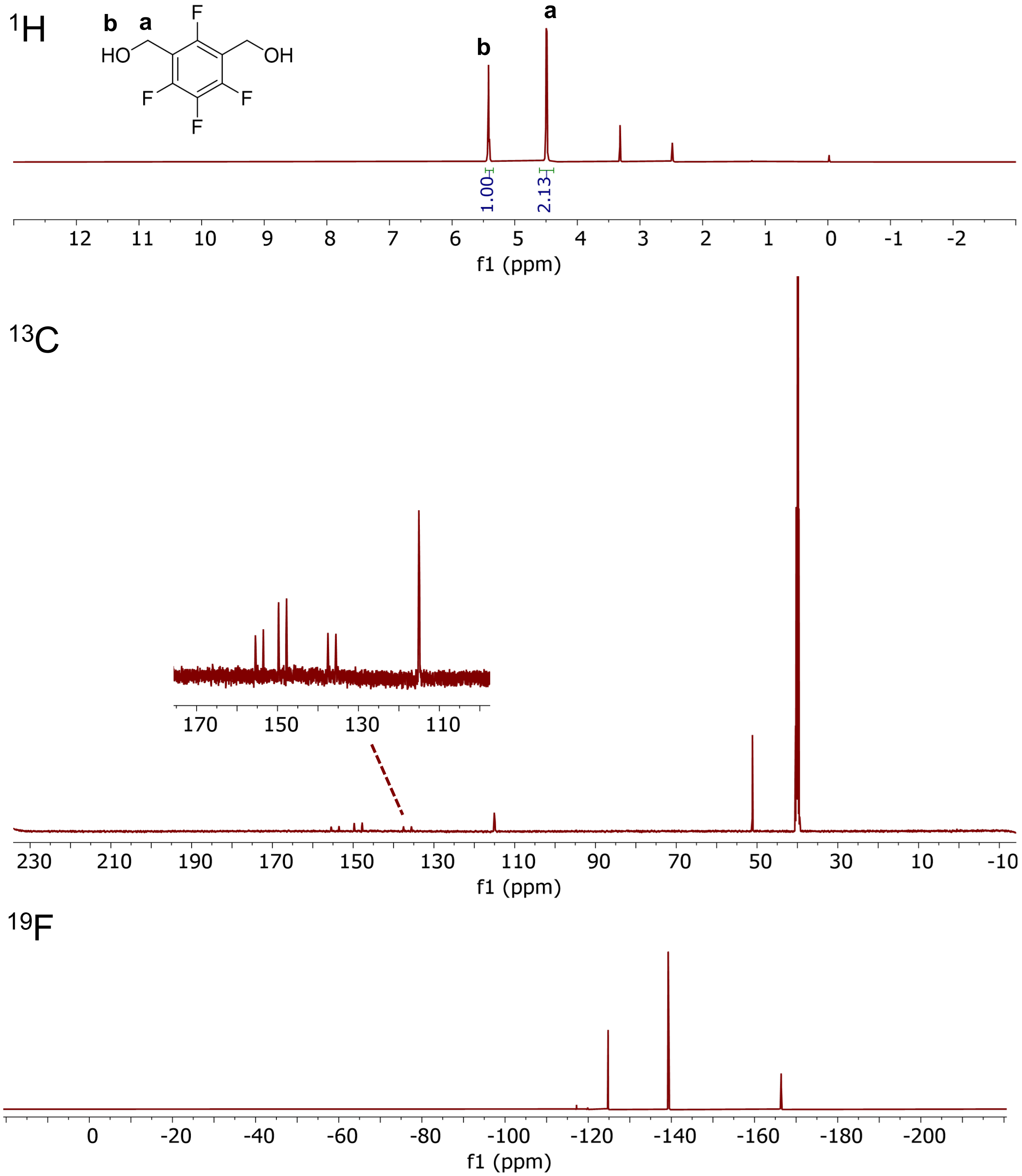

Figure S32. ^{1}H, ^{13}C, and ^{19}F NMR spectra of 13BF.

Table S2. Summary of analysis of synthetic PUs

Sample	Λ (W/(m K)) (averaged at r.t., the error is considered as 0.01 if smaller than 0.005)	Density (g/cm^3)	Volumetric heat capacity (MJ/m^3-K) (at 30 °C)	Atomic density ($x10^{29}/m^3$)	Sound velocity (km/s)	Elastic constant (GPa)	Linear thermal expansion coefficient ($x10^{-6}$ /K)
16H-HDI	0.30±0.01	1.11±0.02	1.82	1.07±0.02	2.79	8.66	119±6
15E-HDI	0.21±0.02	1.27±0.02	2.07	1.14±0.02	2.81	10.00	72±2
16F-HDI	0.14±0.01	1.46±0.03	1.86	0.94±0.02	2.09	6.35	90±12
15H-HDI	0.30±0.03	1.18±0.03	2.14	1.13±0.03	2.59	7.95	181±12
15F-HDI	0.16±0.01	1.45±0.02	2.18	0.99±0.02	2.13	6.58	111±12
16H-IPDI	0.16±0.01	1.07±0.02	1.67	1.06±0.02	2.54	6.89	48±1
15E-IPDI	0.18±0.01	1.11±0.03	1.78	1.04±0.03	2.72	8.21	37±1
16F-IPDI	0.13±0.01	1.27±0.02	1.71	0.89±0.01	2.26	6.50	49±2
16H-MDI	0.35±0.01	1.05±0.06	1.50	0.87±0.05	2.52	6.64	59±1
15E-MDI	0.39±0.02	1.23±0.08	1.56	0.96±0.06	2.79	9.61	49±4
16F-MDI	0.18±0.01	1.45±0.03	1.84	0.87±0.02	2.28	7.51	94±9
14BH-HDI	0.29±0.02	1.20±0.06	1.93	1.03±0.05	2.64	8.33	67±2
13BH-HDI	0.24±0.04	1.16±0.03	2.16	1.01±0.02	2.64	8.06	168±32
12BH-HDI	0.22±0.01	1.19±0.02	1.75	1.03±0.02	2.49	7.38	66±2
14BF-HDI	0.27±0.02	1.23±0.02	1.79	0.86±0.02	2.54	7.93	54±10
13BF-HDI	0.21±0.01	1.24±0.03	1.48	0.87±0.02	2.36	6.92	59±5
12BF-HDI	0.19±0.01	1.27±0.03	1.61	0.89±0.02	2.36	7.09	60±2

Table S3. Summary of glass transition temperature (Tg)/melting temperature (Tm) of PUs

Sample	Glass transition temperature (Tg) (°C)	Melting temperature (Tm) (°C)
16H-HDI	/	166.9
15E-HDI	10.2	155.6
16F-HDI	8.8	117.5, 129.0
15H-HDI	5.5	153.2
15F-HDI	4.3	96.7, 105.6 (weak)
16H-IPDI	81.8	/
15E-IPDI	99.2	/
16F-IPDI	81.1	/
16H-MDI	/	180.9
15E-MDI	/	134.6, 163.8
16F-MDI	/	145.3, 174.6, 193.6
14BH-HDI	/	/(not detected < 200)
13BH-HDI	23.9	168.3
12BH-HDI	34.2	/
14BF-HDI	/	/(not detected < 200)
13BF-HDI	54.3	/
12BF-HDI	50.6	/

References

[1] D. G. Cahill," *Analysis of heat flow in layered structures for time-domain thermoreflectance*", *Review of scientific instruments* **2004**, *75*, 5119-5122.

[2] J. Sun, G. Lv, D. G. Cahill," *Frequency-domain probe beam deflection method for measurement of thermal conductivity of materials on micron length scale*", *Review of Scientific Instruments* **2023**, *94*, 014903.

[3] Y. Zhang, Y. Yan, Y. Wang, M. Ai, H. Jiang, L. Wang, X. Zhao, W. Zhang, Y. Li," *Enhanced Energetic Performances Based on Integration with the Al/PTFE Nanolaminates*", *Nanoscale Research Letters* **2018**, *13*, 206.

[4] aC. Pan, K. Kou, Q. Jia, Y. Zhang, Y. Wang, G. Wu, A. Feng," *Fabrication and characterization of micro-nano AlN co-filled PTFE composites with enhanced thermal conductivity: a morphology-promoted synergistic effect*", *Journal of Materials Science: Materials in Electronics* **2016**, *27*, 11909-11916; bW. Li, H. Li, Y.-M. Zhang," *Preparation and investigation of PVDF/PMMA/TiO2 composite film*", *Journal of Materials Science* **2009**, *44*, 2977-2984; cP. Jia, M. Zhang, L. Hu, R. Wang, C. Sun, Y. Zhou," *Cardanol Groups Grafted on Poly(vinyl chloride)—Synthesis, Performance and Plasticization Mechanism*", *Polymers* **2017**, *9*, 621; dS. Villagómez-Salas, P. Manikandan, S. F. Acuña Guzmán, V. G. Pol," *Amorphous Carbon Chips Li-Ion Battery Anodes Produced through Polyethylene Waste Upcycling*", *ACS Omega* **2018**, *3*, 17520-17527.

[5] PubChem, https://pubchem.ncbi.nlm.nih.gov/compound/136181, **2024**.

[6] PubChem, https://pubchem.ncbi.nlm.nih.gov/compound/Hexamethylenedicarbamic-acid, **2024**.
[7] aR. L. Blaine," *Determination of polymer crystallinity by DSC*", *TA123* **2010**; bB. Wunderlich, *Thermal analysis of polymeric materials*, Springer Science & Business Media, **2005**.
[8] *Vol. Retrieved May 19, 2024*, National Center for Biotechnology Information (2024). , **2024**.
[9] F. Ronkay, B. Molnár, D. Nagy, G. Szarka, B. Iván, F. Kristály, V. Mertinger, K. Bocz," *Melting temperature versus crystallinity: new way for identification and analysis of multiple endotherms of poly(ethylene terephthalate)*", *Journal of Polymer Research* **2020**, *27*, 372.
[10] J. P. Lutz, O. Davydovich, M. D. Hannigan, J. S. Moore, P. M. Zimmerman, A. J. McNeil," *Functionalized and Degradable Polyphthalaldehyde Derivatives*", *Journal of the American Chemical Society* **2019**, *141*, 14544-14548.
